# Altered proteome in translation initiation fidelity defective eIF5^G31R^ mutant causes oxidative stress and DNA damage

**DOI:** 10.1038/s41598-022-08857-y

**Published:** 2022-03-23

**Authors:** Anup Kumar Ram, Monalisha Mallik, R. Rajendra Reddy, Amol Ratnakar Suryawanshi, Pankaj V. Alone

**Affiliations:** 1 School of Biological Sciences, National Institute of Science Education and Research Bhubaneswar, P.O Jatni, Khurda, 752050 India; 2 Homi Bhabha National Institute (HBNI), Anushakti Nagar, Mumbai, 400094 India; 3 Clinical Proteomics, DBT-Institute of Life Sciences, Bhubaneswar, Odisha 751023 India

**Keywords:** Molecular biology, Translation

## Abstract

The recognition of the AUG start codon and selection of an open reading frame (ORF) is fundamental to protein biosynthesis. Defect in the fidelity of start codon selection adversely affect proteome and have a pleiotropic effect on cellular function. Using proteomic techniques, we identified differential protein abundance in the translation initiation fidelity defective eIF5^G31R^ mutant that initiates translation using UUG codon in addition to the AUG start codon. Consistently, the eIF5^G31R^ mutant altered proteome involved in protein catabolism, nucleotide biosynthesis, lipid biosynthesis, carbohydrate metabolism, oxidation–reduction pathway, autophagy and re-programs the cellular pathways. The utilization of the upstream UUG codons by the eIF5^G31R^ mutation caused downregulation of uridylate kinase expression, sensitivity to hydroxyurea, and DNA damage. The eIF5^G31R^ mutant cells showed lower glutathione levels, high ROS activity, and sensitivity to H_2_O_2_.

## Introduction

The fidelity of start codon selection is critical for the synthesis of a normal proteome. Defect in the genes involved in the general translation initiation that alters the fidelity of start codon selection could have a pleiotropic effect on cellular function as they change the cellular proteome. However, no studies have been conducted to understand the change in cellular proteome and its effects on cellular physiology caused by the defect in eukaryotic translation initiation factor fidelity. In eukaryotic translation initiation, the AUG start codon is predominantly selected to establish an open reading frame (ORF) to decode the genetic code while scanning the mRNA from 5′ to 3′ direction by the translation initiation machinery. The *Saccharomyces cerevisiae* translation initiation factors critical for the start codon selection consist of eIF2-GTP-Met-tRNA_i_ Ternary complex (TC), eIF1, eIF1A, eIF5, eIF3, and 40S ribosomal subunit together constitute 43S pre-initiation complex (PIC) that is involve in the selection of AUG start codon on the mRNA^1,2^. The mRNA binds to the eIF4F complex through its 5′ m^7^Gppp cap and it is recruited laterally to the PIC by placing the mRNA into the mRNA entry channel results in the formation of 48S initiation complex^3,4^. The factor eIF1A binds to the A-site of the ribosome and promotes mRNA scanning by its C-terminal tail (CTT)^5^. The factor eIF1 binds near P-site to monitors anti-codon:codon interaction and prevents non-AUG codon selection^1,2^. During the scanning for AUG start codon, the N-terminal GTPase activating protein (GAP) domain of eIF5 interacts with the G-domain of eIF2γ subunit to trigger GTP hydrolysis to form GDP and P_i_. Once the AUG start codon enters the P-site of the 40S ribosome, the anti-codon:codon interaction causes major rearrangement in the scanning complex. The eIF1A-CTT interacts with the N-terminal domain of eIF5, whereas the eIF1 moves out of the P-site of the ribosome, causing the Met-tRNA_i_ to move from P_OUT_ to P_IN_ conformation resulting in the selection of AUG codon and release of P_i_ from the eIF2-GDP-P_i_ complex^3,6,7^.

In yeast, the mitochondrial version of glycyl-tRNA synthetase and alanine-tRNA synthetase is translated using UUG and ACG start codons, respectively^8,9^. However, the molecular mechanisms involved in the non-AUG codon selection in these cases are unknown. The mRNA features such as cis-acting long structured 5′ untranslated region (UTR) or the short unstructured 5′ UTR and the trans-acting mRNA recruitment and assisting factors such as eIF4B, eIF4G, PABP, or Ded1p plays an important role in the effective translation of a selective mRNAs^10–14^. However, defects in the assembly of the 48S initiation complex could adversely affect start codon selection and translation efficacy of global mRNA during the scanning process that can alter general proteome. Mutations isolated in some of the initiation factors such as eIF5^G31R^, eIF2β^S264Y^ and eIF2γ^N135D^ cause relax stringency in the recognition of AUG start codon (Suppressor of initiation codon: Sui^−^ phenotype) proposed to be caused due to the premature P_i_ release, intrinsic GTPase reaction and altered tRNAi binding defect, respectively^15–17^. The Sui^−^ mutants can utilize the in-frame or the out-of-frame UUG, CUG, or GUG codons as a translation start site at the 5′ UTR of the mRNA^16^. The dominant-negative eIF5^G31R^ hyper GAP mutant is the strongest Sui^−^ mutation identified that preferentially recognizes UUG as a start codon and shows severe slow growth, whereas in the recessive conditions the eIF5^G31R^ mutation is lethal^18^. This preferential recognition of UUG start from the 5′ regulatory region of the *GCN4* transcript causes repression of *GCN4* expression (general control non-derepressed: Gcn^−^ phenotype)^19^. We hypothesized that the aberrant translation initiation at the upstream UUG (upUUG) codon by the Sui^−^ mutant might change the cellular proteome that adversely affects cellular physiology and severely cause growth defects. To gain insights in the underpinning cause of growth defect by the translation initiation fidelity defective eIF5^G31R^ mutation, we used gel-based proteomics approach 2D-PAGE followed by MALDI-TOF/MS and gel-free labeling approach iTRAQ (isobaric tag relative absolute quantification) along with nLC-MALDI-TOF/MS technique to quantitate differential protein abundance. Our results suggest a change in the proteome of the eIF5^G31R^ mutant that has major implications in DNA damage and oxidative stress.

## Materials and methods

### Yeast strains used in this study

The *Saccharomyces cerevisiae* yeast strains used in this study were: YP823; *Mat α, ura3-52, leu2-3,112, trp1Δ63, GCN2*+^19^. YP824; *Mat α*, *ura3-52*, *leu2-3,112*, *trp1Δ63, GCN2*+, *his4*Δ*::KanMx6*^20^. Ctt1-loxP-Ura3-loxP-Ctt1 cassette was PCR amplified using oligonucleotides oPA1534 and oPA1535 and B4033 vector as a template. This PCR product was transformed into yeast strain YP823 to delete chromosomal *CTT1* gene to give rise to yeast strain YP939; *MATα leu2-3, -112, ura3-52, trpΔ63, GCN2*^+^*, Gal2*^+^*, ctt1∆::Ura3*. The *ctt1∆* in the YP939 strain was confirmed by PCR using oligonucleotides oPA1569 and oPA584.

Plasmids used in this study are listed in Table 1 and oligonucleotides are listed in the Supplementary Table S1.

**Table 1:** Plasmids used in this study.

Sr. no.	Plasmid number	Plasmid name	Type	References
1	A823	pYcplac22	Single copy (s.c)	^21^
2	A838	pYcplac22-eIF5^G31R^	s.c	This study
3	B4033	pUG72	s.c	^22^
4	A308	pYcplac111	s.c	^21^
5	A309	pYcplac33	sc	^21^
6	B1378	pRS425	High copy (h.c)	^23^
7	A1074	pYcplac33-Ura6-lacZ	s.c	This study
8	A1140	pYcplac33-upUUGless Ura6-LacZ	s.c	This study
9	A1141	pYcplac33-Ura6-3xHA	s.c	This study
10	A1142	pYcplac33-upUUGlessUra6-3xHA	s.c	This study
11	A1144	pYcplac33-CTT1-3xHA	s.c	This study
12	A1147	pRS425-Ura6	h.c	This study
13	A1149	pRS425-CTT1	h.c	This study
14	A1170	pRS425SOD1-SOD2	h.c	This study
15	A1311	pYCplac111-YAP1_LacZ	s.c	This study
16	A1312	pYCplac111-YAP1-upTTGless_LacZ	s.c	This study
17	A1340	pYcplac33-upUUG1in-frame_lacZ	s.c	This study
18	A1341	pYcplac33-upAUG1in-frame_lacZ	s.c	This study
19	A1342	pYcplac33-upUUG3in-frame_lacZ	s.c	This study
20	A1343	pYcplac33-upAUG3in-frame_lacZ	s.c	This study
21	A1346	pRS425-YAP1	h.c	This study

#### Cloning

The 2.2 kb eIF5^G31R^ encoding gene was digested from vector A703^20^ using EcoRI-SalI restriction endonuclease (RE) and cloned into pYCPlac22 to generate pYCPlac22-eIF5^G31R^ (A838) vector. The pYcplac33-Ura6-lacZ reporter construct (A1074) was generated as follows—first, the 534 bp of *URA6* promoter and the N-terminal 12 amino acids region was PCR amplified using oligonucleotide combination oPA1005/oPA1006 and cloned into vector pYcplac33 (A309) at HindIII-SalI site. The *LacZ* region and *URA6* 3′ UTR region were PCR amplified using oligonucleotides combination oPA1009/oPA1010 and oPA1007/oPA1008 respectively and cloned at SalI-BamHI and BamHI-KpnI sites. The four upstream UUG codons from the *URA6* 5′ UTR were removed by fusion PCR using oligonucleotides oPA1005, oPA1012, oPA1013, and oPA1011 and cloned at HindIII-SalI site of vector A1074 to generate pYcplac33-upUUGless Ura6-LacZ (A1140) vector. The C-terminal 3xHA tag was introduced into the *URA6* gene by PCR amplification using oligonucleotides oPA1041/oPA1042 and cloned into A1074 or A1140 vector by replacing *LacZ* gene at SalI-BamHI site to generate pYcplac33-Ura6-3xHA (A1141) and pYcplac33-upUUGlessUra6-3xHA (A1142) vectors, respectively. upUUG codon at the − 95 position was put in-frame with the *URA6-LacZ* reporter by PCR amplification using oligonucleotides oPA1005/oPA1011 followed by a second round of PCR amplification using oPA1005/oPA1467 and cloned at HindIII-SalI site of vector A1074 to generate pYcplac33-upUUG1in-frame_lacZ (A1340) vector. The vector A1340 was modified to insert AUG codon at − 95 position by fusion PCR using oligonucleotides oPA1005, oPA1468, oPA1469, and oPA1470 and cloned at HindIII-SalI site to generate pYcplac33-upAUG1in-frame_lacZ (A1341) vector. The upUUG codon at the − 22 position was put in-frame with the *URA6-LacZ* reporter by fusion PCR using oligonucleotides oPA1005, oPA1012, oPA1013, and oPA1470 and cloned at HindIII-SalI site of vector A1074 to generate pYcplac33-upUUG3in-frame_lacZ (A1342) vector. The vector A1342 was modified to insert AUG codon at − 25 position by fusion PCR using oligonucleotides oPA1005 and oPA1471 using A1342 as a template and cloned at HindIII-SalI to generate pYcplac33-upAUG3in-frame_lacZ (A1343) vector. A 1636 bp of *URA6* gene containing promoter and terminator region from genomic DNA was PCR amplified using oligonucleotide oPA1005/oPA1089 and cloned in pRS425 (B1378) vector at HindIII-SalI site to generate pRS425-Ura6 (A1147) vector.

C-terminal 3xHA tag was introduced into the *CTT1* gene as follows—first, the 520 bp *CTT1* 3′UTR region was PCR amplified using oligonucleotides oPA1036/oPA1037 and cloned at BamHI-KpnI sites of pYcplac33 (A309) vector to generate intermediate vector. Using oligonucleotide oPA1034/oPA1040, a C-terminal 3xHA tag was introduced into the promote-ORF region of the *CTT1* gene by PCR amplification and cloned into the intermediate vector at the BamHI-HindIII site to generate pYcplac33-CTT1-3xHA (A1144) vector. The *CTT1* overexpression construct pRS425-CTT1 (A1149) was generated by amplifying the 2716 bp *CTT1* genomic DNA region using oligonucleotide oPA1108/oPA1109 and cloned into high copy pRS425 vector (B1378) at NotI-BamHI site.

The 1478 bp *SOD1* genomic DNA region was PCR amplified using oligonucleotides oPA1173/oPA1174 and the 1722 bp *SOD2* genomic DNA region was PCR amplified using oligonucleotides oPA1175/oPA1176, digested with NotI-BamHI-HindIII restriction endonuclease and cloned at the NotI-HindIII site of the high copy pRS425 vector to generate pRS425SOD1-SOD2 (A1170) vector.

The pYCplac111-YAP1_LacZ reporter construct (A1311) was generated as follows- first, the 544 bp *YAP1* promoter and the N-terminal 8 amino acids region were PCR amplified using oligonucleotide combination oPA1607/oPA1609 and cloned into vector pYcplac111 (A308) vector at SpeI-BamHI site. The *LacZ* region and *YAP1* 3′ UTR region were PCR amplified using oligonucleotides combination oPA1559/oPA1560 and oPA1608/oPA1610 respectively and cloned at BamHI-SalI and SalI-HindIII sites. The six upstream UUG codons from the *YAP1* 5′ UTR were removed using multiple PCR as follows- First, using A1311 vector template and oligonucleotides combination oPA1607/oPA1640, a PCR product was generated that removed upUUG codons from − 63, − 45, and − 14 position. This PCR product was used as a template to amplify and remove upUUG codon from the − 96 and − 91 position using oligonucleotides combination oPA1609, oPA1638, oPA1639, and oPA1607. Using the last PCR product as a template, the upUUG codon at the − 128 position was removed by PCR amplification using oligonucleotides combination oPA1609, oPA1636, oPA1607, and oPA1637. This PCR product was cloned at the SpeI-BamHI site of the A1311 vector to generate pYCplac111-YAP1-upTTGless_LacZ (A1312) vector. The *YAP1* overexpression construct pRS425-YAP1 (A1346) was generated by amplifying 2792 bp *YAP1* genomic DNA region using oligonucleotide oPA1607/oPA1608 and cloned into pRS425 vector at SpeI-HindIII site.

#### 2D-PAGE followed by MALDI-TOF/MS proteomics approach

Three biological replicates of wild type (WT) and eIF5^G31R^ mutant cells were grown till OD_600_ ~ 1.0. The cells were suspended in 2D lysis buffer (7 M Urea, 2 M Thiourea, 65 mM DTT, 2% CHAPS, and a cOmplete Protease Inhibitor tablet) and mechanically lysed by vortexing using acid-washed glass beads (200 μm) at 4 °C. The supernatant containing total cell extract was separated at 13,000×*g* for 30 min at 4 °C and the total proteins were quantitated by Bradford assay. The total proteins (400 μg) were resolved on 18 cm IPG strip pH 3–11 using Ettan IPGphor 3 isoelectric focusing system (GE) under the following conditions; 12 h (h) at 50 V; 30 min at 250 V; 1 h at 1000 V; 1 h at 2500 V; 1 h at 5000 V; 1 h at 8000 V and held at 8000 V until total Vh reached 50,000 Vh. After IEF, the IPG strips were equilibrated for 30 min in a reduction buffer containing 1% DTT in an equilibration buffer (75 mM Tris–Cl pH 8.8, 6 M urea, 30% v/v glycerol, 2% SDS, 0.002% bromophenol blue), and subsequently alkylated for 30 min in an alkylation buffer containing 2.5% Ioda-acetamide in equilibration buffer. After equilibrium, the strips were placed on 12% polyacrylamide gels and immobilized by overlaying 0.5% agarose on the strips. The second-dimensional separation was carried out in the Bio-Rad Protean II xi Cell and electrophoresed at 80 V for 24 h. The gel was stained with Coomassie brilliant blue (CBB), scanned with GS-800 Calibrated Densitometer (Bio-Rad Laboratories), and analyzed using GE Image Master 2D Platinum v7.0 software. The altered spots were excised, followed by in-gel digestion using trypsin (1 µg/µl trypsin in 1:10 enzyme to protein ratio; AB Sciex #4352157) at 37 °C for 16 h. The resultant digested peptides were subjected to MALDI-TOF/MS. First, it was mixed with α-Cyano-4 hydroxycinnamic acid (CHCA) matrix (1:1) and spotted on MALDI plate and acquired the data as described below.

#### Isobaric tag relative absolute quantification (iTRAQ) proteomics approach

Three biological replicates of the WT and eIF5^G31R^ mutant cells were suspended in phosphate-buffered saline pH 7.2 (PBS) and mechanically lysed as described above. The supernatant containing total cell extract was separated at 13,000×*g* for 30 min at 4 °C and the total proteins were quantitated by Bradford assay. Total protein (400 µg) was buffer exchanged with 1 M urea in 0.5 M Triethylammonium bicarbonate (TEAB) solution using 3 kDa Cut-off filter (Millipore # UFC500324) followed by re-quantification using Bradford assay. An aliquot of 75 μg of total protein was treated with 2% SDS and 50 mM of Tris-(2-carboxyethyl) phosphine (TCEP) and incubated at 36 °C for 1 h. The cysteines were blocked with 200 mM Methyl methane thiosulfonate (MMTS) for 10 min at room temperature and then subjected to trypsin digestion (1 µg/µl trypsin in 1:10 enzyme to protein ratio; AB Sciex #4352157) at 37 °C for 16 h. iTRAQ 4-plex reagents (AB Sciex iTRAQ 4-plex kit #4352135) were used to label the resultant peptide digests according to the manufacturer’s instructions. The different isobaric tags labeled peptides were pooled together and subjected to the strong cation exchange chromatogram using the ICAT column (AB Sciex ICAT Kit #4326687). The samples were eluted using 10–350 mM KCl gradient into 12 fractions. These fractions were desalted using C18 tips (Thermo-Pierce #87784) and vacuum dried (Labconco).

#### NanoLC-MALDI TOF/MS

All the fractions were reconstituted in 0.1% trifluoroacetic acid and chromatogram on reverse phase C18 column (Eksigent, 3 um, 120 Å pores, 0.075 × 150 mm) using Ekspert nanoLC Ultra 2D plus system with 5–90% acetonitrile gradient (2 μl/min flow in 68 min). Eluant from each fraction was deposited with α-CHCA matrix at 15 s/spot and spotted onto the MALDI plate with the help of Ekspot MALDI spotter (AB Sciex). The spotted LC-MALDI plates were subjected to 5800 MALDI TOF/TOF (AB Sciex). Refectron mode mass calibrations were performed externally with a calibration mixture (Cal mix TOF/TOF, AB Sciex #4333604), whereas MS/MS mode mass calibration was executed using the fragments of the Glu-fibrinopeptide B precursor ion. MS and MS/MS data were acquired for all the fractions using defined LC-MALDI parameters. Abundant peptides with a precursor mass of 3568 Da with a mass window ± 100 Da were sent for MS/MS with a 0.8 ppm error. Data Dependent Acquisition (DDA) was activated with automatic Collision Induced Dissociation (CID) and automatic MS/MS acquisition. The MS and MS/MS data of the peptides were used for protein identification and peak areas of the iTRAQ reporter ions suggest the relative abundance of the proteins in the respective samples. The schematics of the iTRAQ and nanoLC-MALDI-TOF/MS approach are outlined in Supplementary Fig. S1a.

#### Data processing and analysis

Quantification and identification of protein were performed on ProteinPilot 4.0.8085 Software (AB Sciex) integrated with Mascot search engine version:2.3. The Paragon algorithm in the ProteinPilot software was used for the peptide identification. User-defined search parameters were as follows: (i) Sample type, iTRAQ 4-plex (Peptide Labeled); (ii) Cysteine alkylation, MMTS; (iii) Digestion, Trypsin; (iv) Instrument, MALDI TOF/TOF AB Sciex 5800, (iv) Mass tolerance 100 ppm for precursor ion and 0.8 ppm for daughter ions, (v) Database, *S. cerevisiae* database from Uniprot, SC strain S288C-20160606.fasta and the corresponding reverse sequence (decoy: for false discovery rate (FDR = 2.0 × decoy hit/total_hit) estimation), (vi) Keratin excluded. The peptides were automatically selected by the Pro Group algorithm to calculate the reporter peak area, error factor (EF), and P-value for quantification. Post quantification dataset was auto bias-corrected to eliminate any variation due to unequal mixing while pooling different labeled peptides. The False Discovery Rate (FDR) was estimated using the Proteomics System Performance Evaluation Pipeline (PSPEP) algorithm integrated into the ProteinPilot, and the Global FDR of 5% was used for further analysis. Quantified proteins from both sets were first normalized against the pooled internal control used in both experimental sets. Post normalization, the dataset was filtered based on unused score cut-off 1.3 or more for further analysis from both sets with at least one unique peptide identified. Proteins present in both sets were further tested for statistical significance by performing Student’s t-test, P-value ± 0.05. A cut-off ≥ 1.5-fold and ≤ 0.66-fold were used for differential protein abundance estimation for up-regulation and down-regulation, respectively. The summary of iTRAQ data analysis is outlined in Supplementary Fig. S1b. In addition, the mass spectrometry proteomics data have been deposited to the ProteomeXchange Consortium via the PRIDE^24^ partner repository with the data set identifier PXD029460.

#### Western blotting

Yeast cells were grown till OD_600_ ~ 0.8. The cell pellet was resuspended in PBS and mechanically lysed as described above. The supernatant containing total cell extract was separated by centrifugation at 13,000×*g* for 20 min at 4 °C. Total proteins were quantitated by Bradford assay. Total proteins were separated on SDS-PAGE followed by electro-blotting to a PVDF membrane. The blots were probed using an anti-HA antibody (Sigma #H9658, 1:10,000 dilution) and horseradish peroxidase (HRP) conjugated mouse secondary antibody (Sigma, #A9044). The blot was developed using SuperSignal (Thermo Scientific #34075) and quantitated using EvolutionCapt Solo S 17.00 software.

#### Real-time PCR

Yeast cells were grown till OD_600_ ~ 0.8 in 30 ml culture. The cell pellet was resuspended in 400 μl TRIzol (Ambion #15596026) and mixed with a 1:1 chloroform-isoamyl alcohol mixture. The supernatant containing total RNA was separated by centrifugation at 13,000×*g* for 2 min at 4 °C. The total RNA was precipitated by isopropanol and after 75% ethanol wash, the pellet was suspended in distilled water and treated with DNase-I at 37 °C for 30 min. The reaction was terminated by heat inactivation at 70 °C for 10 min. The total RNA (0.5 μg) was subjected to cDNA synthesis using SuperScript III enzyme (Invitrogen #18080044) and oligonucleotide oPA1024, oPA1102, oPA1114, and oPA1642 for *ACT1, URA6, CTT1,* and *YAP1* genes, respectively, and the reaction was carried at 50 °C for 60 min. The reaction was stopped by heat inactivation at 70 °C for 15 min. The cDNA was diluted fivefold and a 3 μl of cDNA was subjected to real-time quantitative PCR using Power SYBR Green PCR Master Mix (Applied Biosystem #1601516) and BioRAD CFX384 Touch Real-Time PCR Detection System. The oligonucleotides combination oPA1023/oPA650, oPA1101/oPA1102, oPA1113/oPA1114, and oPA1641/oPA1642 were used for PCR amplification of *ACT1, URA6, CTT1*, and *YAP1* cDNA, respectively.

#### ROS assay

Yeast cells were grown till OD_600_ ~ 0.8, cells were washed with PBS, followed by incubation in PBS containing 100 µM H_2_DCFDA dye (Sigma # D6883) and 5 µM MitoSOX Red (Invitrogen #M36008) for 30 min in the dark at 37 °C followed by washing with PBS. Slides were mounted and observed under Leica SP8 confocal microscope using a 63× objective lens with the FITC (Em λ_529_) filter to measure H_2_DCFDA fluorescence and TRITC (Em λ_580_) filters to measure MitoSOX Red fluorescence. The images were quantitated using NIH ImageJ software (1.52p).

#### β-Galactosidase reporter assay

Yeast cells were transformed with appropriate reporter plasmids. Five colonies from each transformant were grown overnight at 30 °C with shaking at 220 rpm in synthetic dextrose (SD) media with appropriate nutrient supplements and grown till OD_600_ ~ 0.8. Cells were harvested by centrifugation at 13,000×*g* for 20 min at 4 °C and resuspended in LacZ buffer (60 mM Na_2_HPO_4_, 40 mM NaH_2_PO4, 10 mM KCl, and 1 mM MgSO_4_, pH 7.0) and cell lysis was prepared as described above. Clarified cell extract (~ 30 μg) was mixed with LacZ buffer, followed by addition of 180 μl of ONPG (4 mg/ml in LacZ buffer). After 30 min of incubation, absorbance was measured at 420 nm (Molecular Devices Spectra Max). Protein estimation was done using Bradford assay and the β-galactosidase activity (nmol of O-nitrophenyl-β-d-galactopyranoside cleaved per min per mg) was calculated after normalization with total protein.

#### Estimation of cellular glutathione

Yeast cells were grown till OD_600_ ~ 0.8. The cells were washed with PBS and treated with 30 μM monochlorobimane (mCBM) dye for 30 min at 30 °C. Fluorescence adduct formation was imaged at Ex λ_350_ and Em λ_460_ using a Leica SP8 confocal microscope at × 63 magnification. The images were quantitated using NIH ImageJ software (1.52p).

#### Tunel assay

Tunel assay was performed using DeadEnd Fluorometric Tunel System (Promega #G3250). Yeast cells OD_600_ ~ 0.8 (100 μl) was spun down at 4000×*g* for 2 min, followed by fixation in 3.7% formaldehyde for 30 min at room temperature. Cells were washed with PBS and treated with 10 U of lyticase in PBS at 37 °C for 60 min. The cells were washed with PBS and permeabilized using 0.1% triton-X-100 in 0.1% sodium citrate for 5 min on ice. These cells were equilibrated with rTDT and nucleotide mix in 45 μl of equilibration buffer followed by incubation at 37 °C for 1 h. The cells were washed three times with PBS, followed by DAPI staining for 15 min in the dark. The cells were washed with PBS and imaged using Leica SP8 confocal microscope using DIC, DAPI (Em λ_461_) filter and FITC (Em λ_529_) filter. The images were quantitated using NIH ImageJ software (1.52p).

## Results

### Translation fidelity defective eIF5^G31R^ Sui^−^ mutant alters cellular proteome

**Figure 1:**
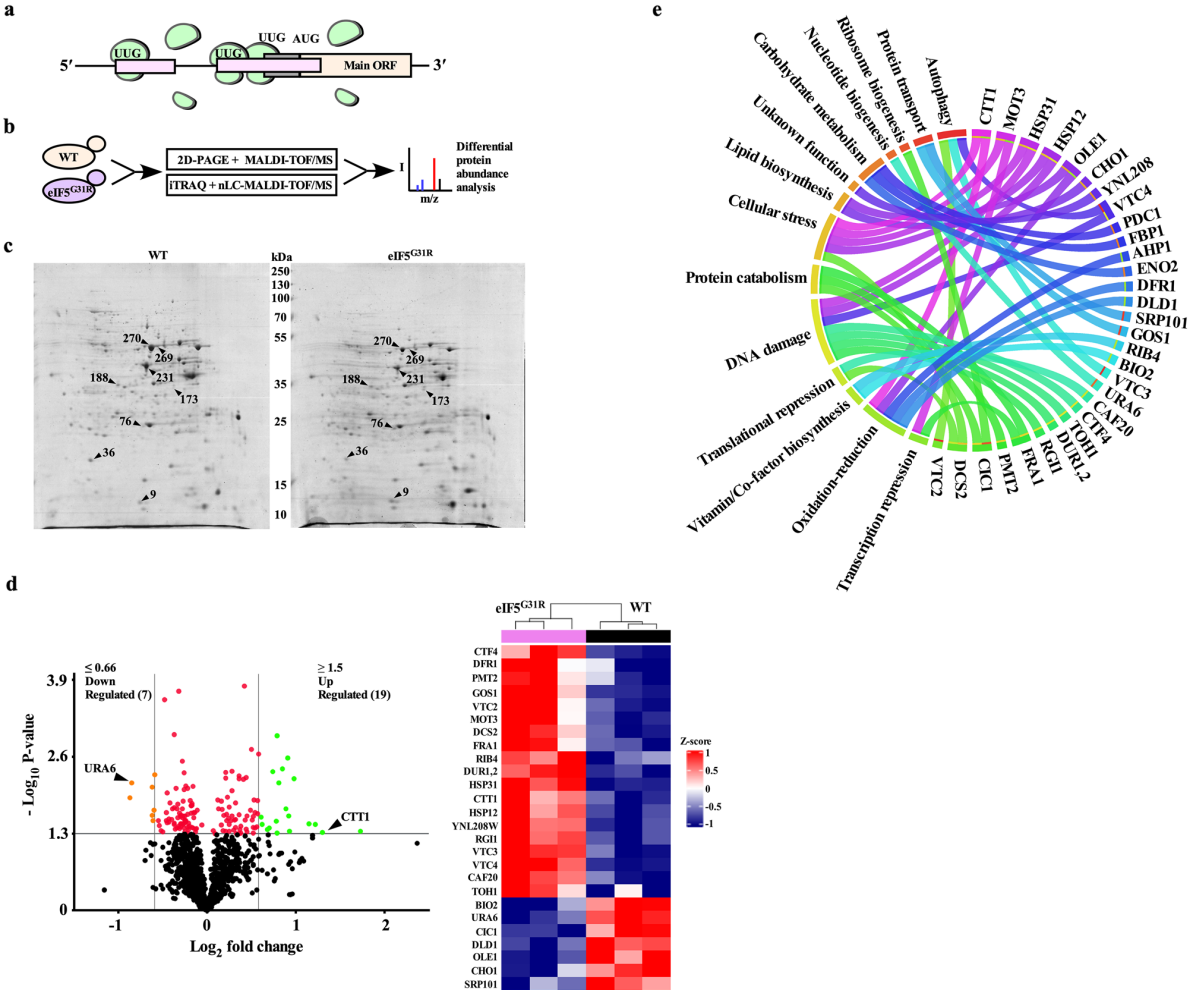
Translation fidelity defective eIF5^G31R^ Sui^−^ mutant alters cellular proteome. (**a**) Schematic of translation of a representative mRNA having upstream ORF initiated from UUG start codon. (**b**) Schematic of the workflow showing different approaches to estimate differential abundance of proteins in the eIF5^G31R^ mutant cells. (**c**) Analysis of altered protein expression by 2D-PAGE. The total cell extract (400 μg of protein) of WT or the eIF5^G31R^ mutant was first electrophoresed on an 18 cm 3–11 IEF strip followed by the second dimension run on 12% SDS-PAGE and stained with CBB. Differentially expressed protein spots were analyzed by GE Image Master Platinum software and subjected to MALDI-TOF for identification. The protein molecular weight marker is shown in the middle. (**d**) Analysis of Isobaric Tag Relative Absolute Quantification (iTRAQ). Total cell extract from WT or eIF5^G31R^ mutant cells was subjected to Isobaric Tag Relative Absolute Quantification (iTRAQ) followed by Mass spectrometric analysis as outlined in Supplementary Fig. S1a. Volcano plot showing − log_10_ (p-value) versus the log_2_ (fold change), where the orange and green dots represent down-regulation (< 0.66 fold cut-off) and upregulated (> 1.5-fold cut-off) proteins, respectively. An arrowhead shows a data-point indicating the expression level of *URA6* and *CTT1* proteins. Heat map showing the expression pattern for all the 26 significantly altered genes in three biological replicates of WT and eIF5^G31R^ mutant cells. (**e**) Analysis of gene function relationship. The association of genes with different biological functions that are altered in the eIF5^G31R^ mutant cells is represented by Chord diagram^64^. Statistical differences were determined by the two-tailed Student’s t-test.

Historically suppressor of initiation codon (Sui^−^) mutations were identified by performing genetic screening to check the growth of *Saccharomyces cerevisiae his4-303* mutant (AUG start codon of *HIS4* gene was mutated to AUU codon) on histidine minus auxotrophic media. The Sui^−^ mutant could utilize the third in-frame UUG codon as an initiation codon and grow on histidine minus medium^15,16^. Our bioinformatic analysis suggests ~ 44.6% of *Saccharomyces cerevisiae* CDS contain at least one UUG codon in the 5′ untranslated region (UTR) within 50 bp of + 1AUG start codon (Supplementary Sheet 1). If Sui^−^ mutants can utilize these upstream UUG codons (upUUG) as translation initiation codon while scanning the mRNA from 5′ to 3′ directions, then that would lead to the formation of either short upORF (ORF with UUG start codon), upORF overlapped out-of-frame with the main ORF or upORF in-frame with main ORF (N-terminal extension) (Fig. 1a). Translation from these out-of-frame upORFs may preclude the translation of main ORF, resulting in the altered proteome in Sui^−^ mutant cells. The eIF5^G31R^ mutation is one of the strongest Sui^−^ mutants that preferentially recognize UUG as a start codon, unlike other Sui^−^ mutants that can also utilize CUG or GUG as a start codon^16,18^. The eIF5^G31R^ mutation in the recessive condition is lethal, whereas the dominant eIF5^G31R^ mutation represses *GCN4* translation (Gcn^−^ phenotype) and shows 3-amino-1,2,4-triazole (3AT) sensitivity due to utilization of upUUG codons from the 5′ regulatory region of *GCN4* transcript and show severe slow growth phenotype^19,20^. To understand how strong utilization of the upUUG codon by the eIF5^G31R^ mutation alters the cell's proteome, we employed a gel-based approach 2D-PAGE followed by MALDI-TOF/MS and a gel-free labeled approach iTRAQ 4-plex coupled with nLC-MALDI-TOF/MS to quantitate the differential abundance of proteins (Fig. 1b). Whole cell extract (WCE) from the WT and eIF5^G31R^ mutant was resolved on 2D-PAGE and differentially abundance proteins spots were analyzed using GE Image Master Platinum software (Fig. 1c). Significantly altered protein spots (total 16) were subjected to LC-MALDI-TOF/MS analysis. Total four proteins could be successfully identified as summarized in Table 2. Four proteins abundance downregulated in the eIF5^G31R^ mutant are involved in the glycolysis pathway and antioxidant activity (Supplementary Table S3). A total of 1098 common proteins (Supplementary Fig. S1b) was successfully identified using a gel-free iTRAQ labeled approach with a significant score in both experimental sets, of which seven proteins abundance were downregulated (< 0.66-fold cut-off), while nineteen proteins abundance were upregulated (> 1.5-fold cut-off) (Fig. 1d, Table 3). Categorizing these proteins for their cellular function and gene ontology indicates involvement in DNA replication stress (*VTC4, HSP12, RGI1, HSP31, DUR1,2, TOH1, CTF4*)^25^, micro-autophagy (*VTC2, VTC3, VTC4*)^26^, protein oxidation/degradation pathway (*PMT2, CIC1, FRA1*)^27–29^, cellular stress (*MOT3, DCS2, CTT1, HSP31, HSP12*), translation repression *(CAF20, MOT3, DCS2)*^30–34^, lipid biosynthesis (*CHO1, OLE1*)^35^, oxidation–reduction pathway (*DLD1, OLE1, DFR1, CTT1*)^36–38^, transcription regulation *(FRA1, MOT3*)^28,39^, vitamin/co-factor biosynthesis (*RIB4, BIO2*)^40,41^, ribosome biogenesis (*CIC1*)^42^, protein transport (*GOS1, SRP101*)^43,44^, pyrimidine nucleotide biosynthesis gene *(URA6)*^45^ and one uncharacterized protein of unknown function *(YNL208W)* (Supplementary Table S3, Fig. 1e).

**Table 2:** List of altered protein spots identified by gel-based 2D-PAGE/MALDI-TOF/MS approach.

Sr. no.	Accession # (UniProt database)	Spot ID	Gene	Fold change (eIF5^G31R^/WT)	P-value	Actual mol. wt (kDa)	On gel approx. mol. wt (kDa)
1	P00925	270	ENO2	0.3448	0.0456	47	47
2		76	ENO2	2.0886	0.0191	47	24
3		269	ENO2	0.5060	0.0009	47	47
4	P14540	231	FBP1	0.6040	0.0401	38	38
5	P38013	36	AHP1	0.6047	0.0370	19	19
6	P06169	9	PDC1	1.6507	0.0026	61	13
7		173	PDC1	2.6654	0.0299	61	34
8	P14540	188	FBP1	1.7153	0.0067	38	34

**Table 3:** List of altered proteins identified by gel-free labeled iTRAQ along with nLC-MALDI-TOF/MS approach.

Sr. no.	Accession # (UniProt database)	Gene	Fold change (eIF5^G31R^/WT)	P-value
1	P21147	OLE1	0.5477	0.0124
2	P15700	URA6	0.5546	0.0069
3	P32451	BIO2	0.6493	0.0081
4	P08456	CHO1	0.6500	0.0246
5	P32916	SRP101	0.6570	0.0304
6	P38779	CIC1	0.6613	0.0200
7	P32891	DLD1	0.6661	0.0050
8	P50861	RIB4	1.5247	0.0261
9	P31382	PMT2	1.5368	0.0344
10	P07807	DFR1	1.5977	0.0424
11	Q07825	FRA1	1.6199	0.0396
12	P40043	RGI1	1.6674	0.0044
13	Q01454	CTF4	1.7217	0.0309
14	P46992	TOH1	1.7233	0.0489
15	P32528	DUR1,2	1.7275	0.0011
16	Q02725	VTC3	1.7513	0.0068
17	P47075	VTC4	1.8045	0.0040
18	P40159	YNL208W	1.8591	0.0191
19	Q04432	HSP31	1.8778	0.0026
20	P22943	HSP12	1.8912	0.0252
21	P43585	VTC2	1.9087	0.0453
22	P12962	CAF20	1.9685	0.0059
23	Q12123	DCS2	2.2230	0.0340
24	P38736	GOS1	2.3314	0.0344
25	P06115	CTT1	2.4573	0.0475
26	P54785	MOT3	3.3204	0.0459

#### Upstream UUG codons repress the *URA6* expression in the eIF5^G31R^ mutant and cause DNA damage

**Figure 2:**
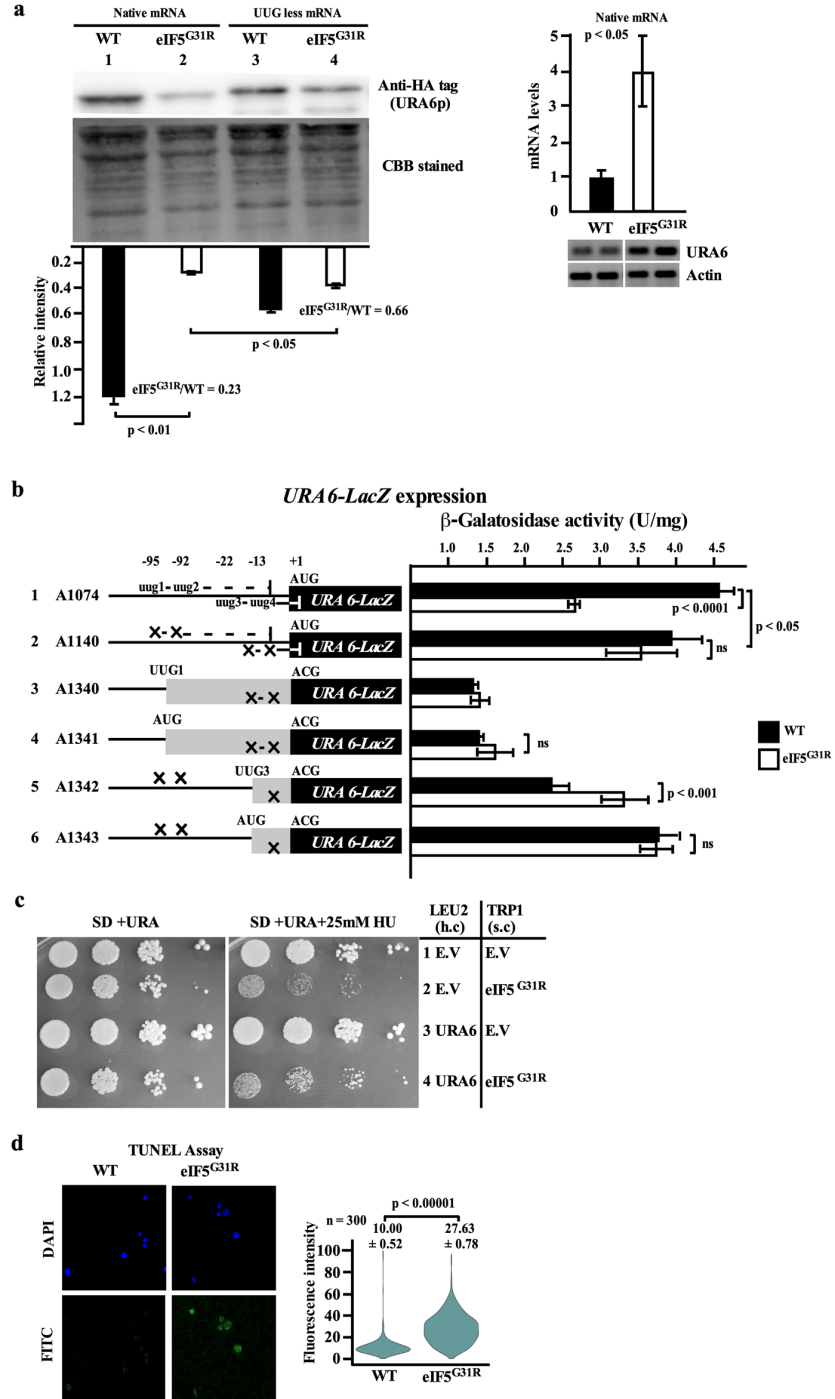
Upstream UUG codons repress the *URA6* expression in eIF5^G31R^ mutant and cause DNA damage. (**a**) Quantification of *URA6* protein expression. YP823 strain transformed with pYcplac33-Ura6-3xHA (A1141) or pYcplac33-upUUGlessUra6-3xHA (A1142) constructs along with empty vector pYcplac22 (A823) or pYcplac22-eIF5^G31R^ (A838) construct were grown overnight on synthetic dextrose (SD) plus leucine medium till OD_600_ ~ 0.8 at 30 °C. Whole cell extract (WCE) was prepared by mechanical cell breaking using glass beads and 40 µg of WCE was subjected to 12% SDS-PAGE and the URA6p was identified by Western blot using an anti-HA antibody and normalized with blot stained by Coomassie brilliant blue (CBB) (left). YP823 strain carrying empty vector pYcplac22 (A823) or pYcplac22-eIF5^G31R^ (A838) constructs were grown overnight on synthetic dextrose (SD) plus leucine medium till OD_600_ ~ 0.8 at 30 °C. The whole-cell extract (WCE) was treated with TRIzol, followed by ethanol precipitation. The total RNA isolated was subjected to reverse transcription and quantitative PCR using *URA6* ORF specific oligonucleotides oPA1101 and oPA1102 (right). The error represents an average deviation. (**b**) Analysis of *URA6-LacZ* expression. Yeast strain YP823 containing empty vector pYcplac22 (A823) or pYcplac22-eIF5^G31R^ (A838) constructs were transformed with derivatives of *Ura6-LacZ* reporter constructs A1074, A1140, A1340, A1341, A1342, and A1343 were grown till OD_600_ ~ 0.8 in SD plus leucine medium at 30 °C. WCE were prepared, and β-galactosidase activity (nmol of *O*-nitrophenyl-β-d-galactopyranoside cleaved per min per mg) was measured and plotted. The error represents an average deviation. (**c**) Growth analysis in the presence of hydroxyurea. High copy (h.c) empty vector (EV) pRS425 (B1378) or high copy pRS425-Ura6 (A1147) constructs were transformed in yeast strain YP828 carrying either EV pYcplac22 (A823) or pYcplac22-eIF5^G31R^ (A838) constructs were grown overnight, serially diluted, and spotted on SD plus uracil or SD plus uracil plus 25 mM hydroxyurea and incubated at 30 °C for 2–3 days. (**d**) Analysis of DNA fragmentation by TUNEL assay. Yeast strain YP823 carrying either EV pYcplac22 (A823) or pYcplac22-eIF5^G31R^ (A838) constructs were grown till OD_600_ ~ 0.8. The cells were treated with rTDT and nucleotide mix and imaged using Em λ_461_ (DAPI) and Em λ_529_ (FITC) filters. The values are average (n = 300) along with the standard error of the mean (SEM). Statistical differences were determined by the two-tailed Student’s t-test. *ns* non-significant.

*URA6* is an essential gene that encodes uridylate kinase and takes part in the *de-novo* pyrimidine biosynthesis pathway to catalyze the conversion of uridine monophosphate (UMP) to uridine-5′-diphosphate (UDP). The UDP is further converted into UTP and CTP, which are the precursors for DNA and RNA biosynthesis^46^. Our iTRAQ data analysis showed ~ 0.55-fold less abundant (P-value, 0.0069) uridylate kinase protein (Fig. 1d). To validate this data, we modified the *URA6* gene by adding a C-terminal 3xHA tag and checked the plasmid-borne expression of this modified gene in the WT and eIF5^G31R^ mutant yeast strain by Western blot using Anti-HA tag antibody. Consistent with the iTRAQ data analysis, the Western blot showed ~ 0.23-fold less abundance of uridylate kinase protein (Fig. 2a, left). To check if the lower uridylate kinase protein levels were not due to the lower transcription of its mRNA, we performed RT-qPCR using *URA6* ORF specific oligonucleotides. To our surprise, we observed ~ 3.5-fold higher *URA6* mRNA expression levels in the eIF5^G31R^ mutant, suggesting that the lower levels of uridylate kinase protein indicate translation defect (Fig. 2a, right). It may be possible that the presence of the four upUUG codons at the ~ 160 nucleotides (nt) long 5′ UTR^45^ has generated an out-of-frame upORFs that prematurely terminate translation before reaching the *URA6* main ORF in the eIF5^G31R^ mutant. To test this, we mutated all four upUUG codons (at − 95, − 92, − 22, and − 13 positions) and checked the uridylate kinase protein levels by Western blot. The removal of all upUUG codons caused an overall reduction in uridylate kinase protein levels in the WT cells. However, the relative expression of uridylate kinase protein increased (ratio 0.66) in the eIF5^G31R^ mutant after the removal of upUUG codons from the mRNA as compared to the WT (ratio 0.23) mRNA (Fig. 2a). These results suggest that the eIF5^G31R^ mutant utilizes upUUG codons to translate the out-of-frame upORFs and the ribosomes dissociate before reaching the main ORF causing lower uridylate kinase protein expression.

The utilization of upUUG codons at the 5′ UTR region by the eIF5^G31R^ mutant creates two upORFs. The upUUG1 (− 95) and upUUG2 (− 92) codons are in the same reading frame and constitute upORF1 that terminates before the main ORF of *URA6* gene. The upUUG3 (− 22) and upUUG4 (− 13) codons are in the same reading frame and constitute the second upORF2 that partially overlaps out of frame with the main *URA6* ORF (Fig. 2b). To probe which upUUG codons are utilized by the eIF5^G31R^ mutant, we made the *URA6-LacZ* reporter construct (A1074). This construct was modified to remove all upUUG1-4 codons (A1140), removal of upUUG3-4 and keeping upUUG1 intact in-frame with *URA6-LacZ* (A1340), removal of upUUG3-4 and changing upUUG1 into AUG codon in-frame with *URA6-LacZ* (A1341), removal of upUUG1, 2, and 4 and keeping upUUG3 intact in-frame with *URA6-LacZ* (A1342), and removal of upUUG1, 2 and 4 and changing upUUG3 to AUG codon in frame with *URA6*-*LacZ* (A1343). These constructs were transformed into the WT or eIF5^G31R^ mutant yeast cells to check the resultant β-galactosidase activity. Consistent with the results obtained with Western blot, the removal of all the upUUG codons increased the expression of *URA6-LacZ* reporter in the eIF5^G31R^ mutant. Moreover, whereas the translation from the − 95 position (upUUG1 or AUG) codon severely down-regulated reporter expression, the translation from the − 22 position (upUUG3 or AUG) showed better reporter expression (Fig. 2b). These results suggest that eIF5^G31R^ mutant initiated translation from the second upORF2 that partially overlaps out-of-frame with the main *URA6* ORF by utilizing the upUUG3 codon.

If the uridylate kinase protein levels are downregulated in the eIF5^G31R^ mutant strain, it should also affect the catalysis of UMP to UDP. The UDP is further converted into UTP and CTP. Since the UTP and CTP are the precursors for DNA and RNA biosynthesis, we propose that the eIF5^G31R^ mutant affects nucleic acid biosynthesis. Our iTRAQ data analysis showed a significant abundance of proteins previously reported to be associated with DNA replication stress (*TDH2, VTC4, PRB1, HSP31, HSP12, TOH1, DUR1,2 & RGI1*)^25^. Yeast cells under DNA replication stress show sensitivity to hydroxyurea (HU). To check if the eIF5^G31R^ mutation is sensitive to DNA replication stress, we checked the growth on 25 mM HU. The eIF5^G31R^ mutation was sensitive to HU and the sensitivity was partially rescued by a high copy overexpression of *URA6* gene (Fig. 2c). The DNA replication stress also induces DNA damage that can be tested by a TUNEL assay. Consistently, the eIF5^G31R^ mutant cells showed a twofold increase in the TUNEL positive cells (Fig. 2d).

#### eIF5^G31R^ mutation causes oxidative stress

**Figure 3:**
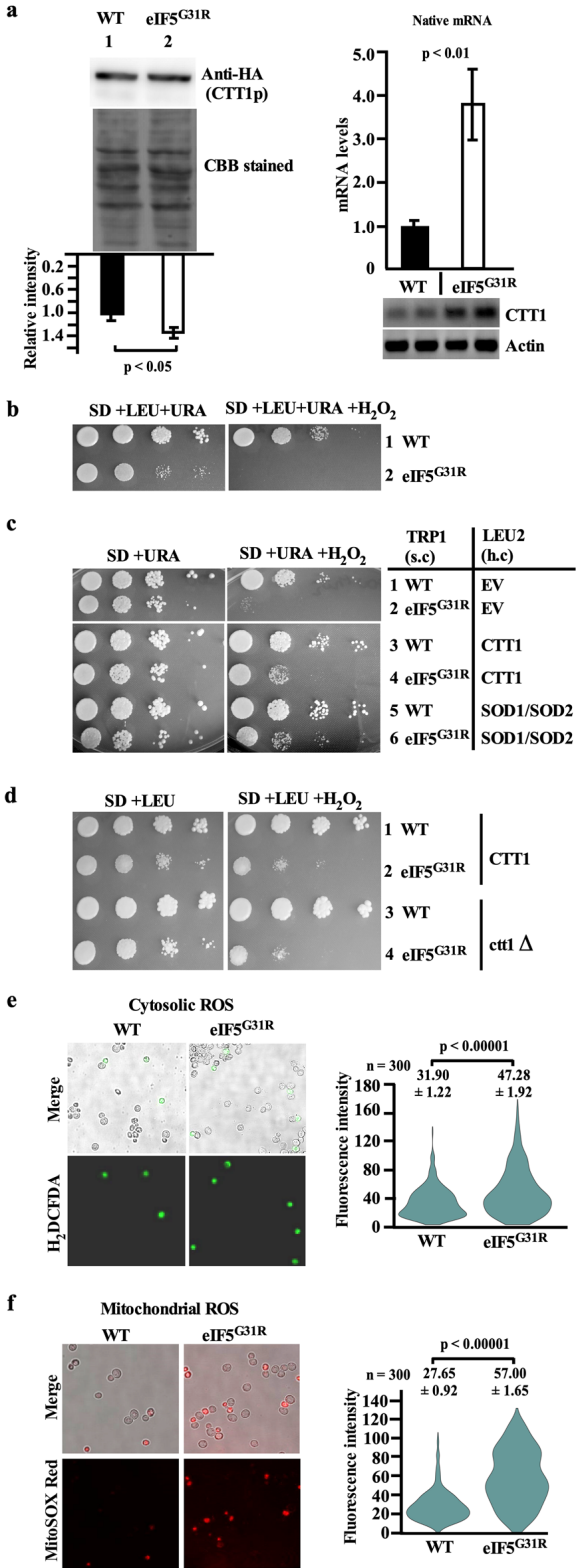
eIF5^G31R^ mutant causes oxidative stress. (**a**) Quantitation of *CTT1* expression. Yeast strain YP823 transformed with pYcplac33-CTT1-3xHA (A1144) construct along with an empty vector pYcplac22 (A823) or pYcplac22-eIF5^G31R^ (A838) constructs were processed as Fig. 2a. CTT1p was identified by Western blot using an anti-HA antibody and normalized with blot stained by CBB (left). The *CTT1* mRNA was quantitated by RT-qPCR using *CTT1* ORF specific oligonucleotides oPA1113 and oPA1114 (right) as described in Fig. 2a (right). The error represents an average deviation. (**b–d**) Growth analysis in the presence of H_2_O_2_. Yeast strain as in Fig. 2c was serially diluted and spotted on SD, leucine, uracil or SD, leucine, uracil plus 3 mM H_2_O_2_ plates and incubated at 30 °C for 2 days (**b**). Yeast strain YP823 carrying EV pYcplac22 (A823) or pYcplac22-eIF5^G31R^ (A838) constructs were transformed with either high copy EV pRS425 (B1378), pRS425-CTT1 (A1149)*,* pRS425SOD1-SOD2 (A1170) constructs were grown overnight, serially diluted, and spotted on SD, uracil or SD, uracil plus 3 mM H_2_O_2_ plates (**c**). Yeast strain YP939 (*ctt1*∆) and its isogenic WT strain (YP823) were transformed with EV pYcplac22 (A823) or pYcplac22-eIF5^G31R^ (A838) constructs were grown overnight, serially diluted, and spotted on SD, leucine or SD, leucine plus 2 mM H_2_O_2_ plates (**d**). (**e,f**) Analysis of oxidative stress. Yeast strain YP823 was transformed with either EV pYcplac22 (A823) or pYcplac22-eIF5^G31R^ (A838) constructs were grown in SD plus leucine, uracil medium till OD_600_ ~ 0.8 at 30 °C. 200 μl cells suspension were stained with either 100 μM of H_2_DCFDA to check cytosolic ROS or 5 μM of MitoSOX Red to check mitochondrial ROS. Fluorescence microscopic imaging was performed using FITC (Em λ_529_) filter to measure H_2_DCFDA fluorescence and TRITC (Em λ_580_) filters to measure MitoSOX Red fluorescence. The values are average (n = 300) along with the standard error of the mean (SEM). Statistical differences were determined by the two-tailed Student’s t-test.

The catalase-T enzyme (*CTT1*) showed higher abundance in the eIF5^G31R^ mutant as per our iTRAQ analysis. The higher abundance of the catalase-T enzyme indicates oxidative stress in the eIF5^G31R^ mutant cells. To test this, we first checked the levels of catalase-T by adding a C-terminal 3xHA tag and analyzed the plasmid-borne expression of this modified gene in the WT and eIF5^G31R^ mutant yeast strain by Western blot using anti-HA tag antibody. Consistent with the iTRAQ data analysis, the Western blot showed 1.3-fold upregulation of catalase-T protein (Fig. 3a, left). We also checked the transcription level of *CTT1* mRNA by RT-qPCR using *CTT1* ORF specific oligonucleotides. There was ~ threefold higher expression of *CTT1* mRNA in the eIF5^G31R^ mutant cells (Fig. 3a, right). The discrepancy between the *CTT1* mRNA expression and protein expression could be because of the presence of the four upUUG codons (− 105, − 76, − 66 and − 40) in the 112 nucleotides long 5′ UTR of *CTT1* mRNA. The upUUG codons at the − 105 and − 66 position are in-frame with the main AUG start codon, whereas the upUUG codon − 76 and − 40 are out-of-frame with the main AUG start codon. Since the upUUG codon at the − 105 position is very close to the 5′ cap of the mRNA and could be skipped by the eIF5^G31R^ mutant containing 48S scanning ribosome. However, the eIF5^G31R^ mutant could initiate translation from the out-of-frame − 76 or − 40 upUUG codon causing premature termination, whereas initiation from the in-frame upUUG codon at − 66 position could result in translation of the catalase-T protein. Taken together, this results in ~ 1.3-fold increase in the catalase-T protein expression as the *CTT1* mRNA levels are high. These results suggest eIF5^G31R^ mutant cells must be under oxidative stress and in response to this stress, the expression of the *CTT1* transcript could have been upregulated. The yeast cells under oxidation stress should show sensitivity to H_2_O_2_. Consistently, the eIF5^G31R^ mutant showed strong sensitivity to H_2_O_2_ treatment (Fig. 3b). The sensitivity to oxidative stress was partially rescued in the eIF5^G31R^ mutant cells when the *CTT1* or *SOD1/SOD2* genes were overexpressed from a high copy plasmid (Fig. 3c). Moreover, the oxidative stress was exacerbated in the eIF5^G31R^ mutant cells when the *CTT1* gene was deleted (Fig. 3d). The oxidative stress in the eIF5^G31R^ mutant may have been triggered by the accumulation of reactive oxygen species (ROS) such as hydrogen peroxide (H_2_O_2_), the hydroxyl radicals (OH^·^), and the superoxide anion (O_2_^·−^). To check if the eIF5^G31R^ mutant has higher ROS levels, we treated the eIF5^G31R^ mutant cells with H_2_DCFDA and MitoSOX red dye to check cytosolic and mitochondrial ROS levels, respectively. The 1.48-fold high fluorescence for H_2_DCFDA and 2.06-fold high fluorescence for MitoSOX red suggests high ROS levels in the eIF5^G31R^ mutant cells (Fig. 3e,f).

#### Lower levels of glutathione and YAP1 gene expression exacerbate the oxidative stress in the eIF5^G31R^ mutant

**Figure 4:**
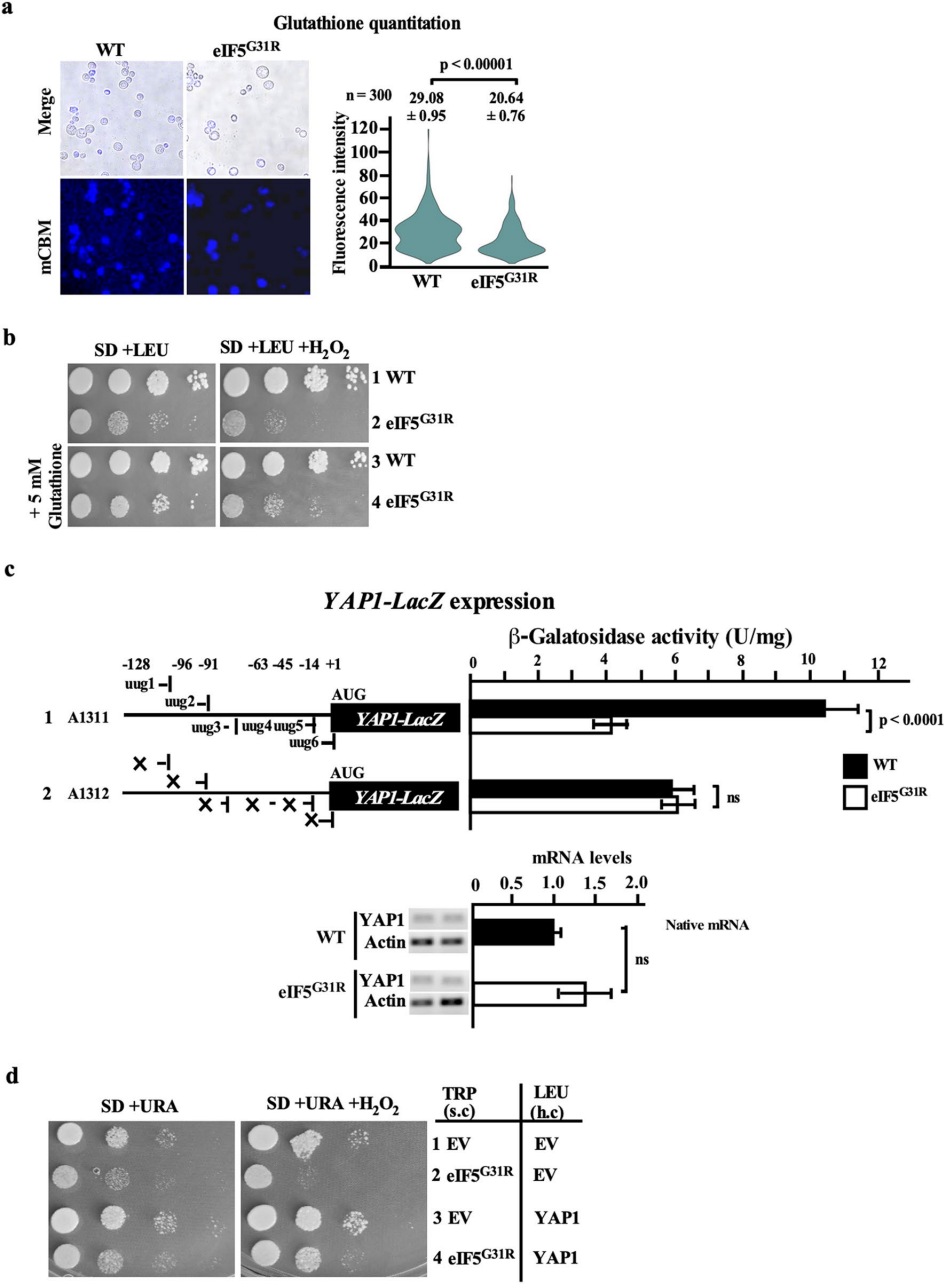
Lower level of glutathione and *YAP1* gene expression exacerbates oxidative stress in eIF5^G31R^ mutant cells. (**a**) Glutathione quantification. Yeast strain YP823 carrying EV pYcplac22 (A823) or pYcplac22-eIF5^G31R^ (A838) constructs were grown in SD plus leucine, uracil medium till OD_600_ ~ 0.8 at 30 °C. 200 μl cells suspension were stained with 30 μM of monochlorobimane (mCBM) followed by fluorescence microscopic imaging at Em λ_460_. The values are average (n = 300) along with the standard error of the mean (SEM). (**b**) Growth analysis in the presence of glutathione. Yeast strains as per (**a**) were spotted on either SD plus leucine, uracil plus 2 mM H_2_O_2_ or supplemented with 5 mM glutathione plates and incubated at 30 °C for 3 days. (**c**) Quantification of *YAP1-LacZ* expression. Yeast strain YP823 carrying EV pYcplac22 (A823) or pYcplac22-eIF5^G31R^ (A838) constructs were transformed with pYCplac111-YAP1_L*acZ* (A1311) or pYCplac111-YAP1-upTTGless_LacZ (A1312) construct, were grown till OD_600_ ~ 0.8 at 30 °C and the β-galactosidase activity was measured as per Fig. 2b. The error represents an average deviation. (**d**) Growth analysis in the presence of H_2_O_2_. Yeast strain YP823 carrying EV pYcplac22 (A823) or pYcplac22-eIF5^G31R^ (A838) constructs were transformed with either high copy EV pRS425 (B1378) or (h.c) pRS425-YAP1 construct (A1346), grown overnight, serially diluted, and spotted on SD, uracil plus 2 mM H_2_O_2_ as per Fig. 3c. Statistical differences were determined by the two-tailed Student’s t-test. *ns* non-significant.

Our data showed higher levels of ROS in the eIF5^G31R^ mutant cells. Catalase, superoxide dismutase, glutathione peroxidase, and thioredoxin system neutralize the intracellular ROS^47–49^. Our iTRAQ data analysis showed 20% downregulation of GSH1 protein levels in the eIF5^G31R^ mutant (Supplementary Sheet 2), suggesting that there may be inadequate levels of glutathione to detoxify the cellular ROS. We check the intracellular glutathione levels by monochlorobimane dye, which reacts with glutathione in the presence of glutathione-S-transferase to form an adduct that shows fluorescence at 380 nm^50,51^. Microscopic image analysis showed a 30% reduction of glutathione level in the eIF5^G31R^ mutant cells (Fig. 4a). The growth defect associated with the eIF5^G31R^ mutant was partially rescued in the presence of the 5 mM glutathione (Fig. 4b). However, the high copy overexpression of *GSH1* and *GSH2* genes did not rescue the slow growth of the eIF5^G31R^ mutant (data not shown). It could be possible that the other components of the redox system critical for ROS detoxification are not fully expressed in the eIF5^G31R^ mutant cells.

*YAP1* is a AP-1 family transcription factor that regulates ~ 28 genes associated with ROS detoxification, including *GSH1, GSH2, GLR1, SOD1, SOD2, GPX2, TSA1, TRR1, AHP1, and TRX2*. These genes are involved in glutathione biosynthesis, superoxide dismutase activity, glutathione peroxidase function, thioredoxin, and thioredoxin peroxidases action^52^. The YAP1 protein was not identified in our iTRAQ or 2D-PAGE data analysis. However, analysis of the *YAP1* gene transcript suggests the presence of six out-of-frame upUUG codons at the 5′ UTR that may affect its translation. We made *YAP1-LacZ* reporter constructs with native 5′ UTR region containing the six upUUG codons (A1311) and the modified construct devoid of the six upUUG codons (A1312) and these constructs were transformed in the WT or eIF5^G31R^ mutant cells. The eIF5^G31R^ mutant showed 2.4-fold less YAP1 reporter expression from the native constructs. However, upon removing six upUUG codons, the reporter expression in the eIF5^G31R^ mutant was similar to the WT (Fig. 4c). This result suggests that the YAP1 protein expression was downregulated in the eIF5^G31R^ mutant, which may have affected the transcription of the target genes involved in the redox pathway. Consistently, we observed down-regulation of the AHP1 mRNA (Supplementary Fig. S6) and AHP1 protein levels that protect against oxidative damage^53^ (Table 2). However, overexpression of the *YAP1* gene from a high copy plasmid should increase the YAP1 protein levels and show a detoxifying effect on ROS. Consistently, the YAP1 overexpression showed partial growth rescue of the eIF5^G31R^ mutant in the presence of H_2_O_2_ (Fig. 4d).

## Discussion

Previously, we reported that the eIF5^G31R^ mutation recognizes UUG codon but does not initiate translation from CUG or GUG codon^18^. In this study, we decipher the underpinning effect of the eIF5^G31R^ mutation on UUG codon recognition that alters cellular proteome in dominant conditions. Our iTRAQ data suggests that seven proteins abundance was downregulated, while nineteen proteins abundance was upregulated. The most striking of them was the 0.55-fold downregulation of the uridylate kinase protein (*URA6*), which takes part in the de-novo pyrimidine biosynthesis pathway. This downregulation of uridylate kinase was not due to the lower levels of *URA6* transcript; rather, our analysis suggests that the utilization of the upstream UUG codons could generate out-of-frame upORFs that impedes translation initiation from the *URA6* main ORF. Removal of all the four upUUG codons from the *URA6* transcript caused an overall reduction in the uridylate kinase translation in the WT cells. We suspect mutating the upUUG codons may have changed the secondary structure of the mRNA resulting in the lower translation of the *URA6* transcript. However, the relative expression of uridylate kinase increased in the eIF5^G31R^ mutant cells after removing the four upUUG codons (Fig. 2a). The eIF5^G31R^ mutant cells could have unsuccessfully tried to mitigate the low abundance of uridylate kinase by upregulating the *URA6* mRNA expression to relieve the lower nucleotide availability stress. The upregulation of DNA replication stress markers (*TDH2, VTC4, PRB1, HSP31, HSP12, TOH1, DUR1,2 & RGI1*)^25^ observed in our iTRAQ data is consistent with the sensitivity of the eIF5^G31R^ mutant to hydroxyurea because of the low uridylate kinase expression and the attendant DNA damage (Fig. 2c,d).

The upregulations of catalase-T in the eIF5^G31R^ mutant proteome suggest oxidative stress. Consistently, the eIF5^G31R^ mutant showed sensitivity to H_2_O_2_, which was partially rescued by the high copy overexpression of either *CTT1* or *SOD1/SOD2* genes (Fig. 3b,c). The sensitivity to H_2_O_2_ is consistent with the high cytosolic and mitochondrial ROS levels in the eIF5^G31R^ mutant (Fig. 3e,f). Compounding this oxidative stress, glutathione levels were 30% lower in the eIF5^G31R^ mutant cells (Fig. 4a), and supplementing 5 mM glutathione could partially rescue its growth defect (Fig. 4b). However, we could not identify a major source of ROS generation in the eIF5^G31R^ mutant through our proteome analysis. Previous report from Salmon and co-workers, suggest that the DNA damage alone can elicit an increase in the intracellular ROS levels^54^. Thus the DNA damage observed in the eIF5^G31R^ mutant (Fig. 2d) could trigger an intracellular ROS generation, and the components involved in ROS detoxification were not optimum due to the downregulation of regulatory genes, thus tipping the balance towards oxidative stress. *YAP1* is an AP-1 family transcription factor that regulates ~ 28 genes associated with ROS detoxification^52^ and the *YAP1-LacZ* reporter expression was observed to be downregulated in the eIF5^G31R^ mutant because of the upUUG codon utilization. Consistently, the overexpression of the *YAP1* gene partially rescued the sensitivity to oxidative stress (Fig. 4c,d).

Our results indicate that the defect in translation initiation fidelity in the eIF5^G31R^ mutant re-programs the cellular pathways to cause DNA damage and oxidative stress. Moreover, the upregulation of proteins involved in the protein degradation and micro-autophagy pathways suggests an enhanced turnover of proteins in the eIF5^G31R^ mutant cells. Consistently, we observed enhanced degraded products of FBP1, PDC1 and ENO2 proteins in 2D-PAGE MALDI-TOF/MS analysis (Table 2). We did not observe significant changes in the 40S, 60S, and 80S ribosomal (monosome) peaks in the eIF5^G31R^ mutant (Supplementary Fig. S2), suggesting that the mutation may not have affected the general assembly of PIC during the translation initiation process and only affected the 5′ to 3′ mRNA scanning where the eIF5^G31R^ mutation preferentially selected UUG as initiation codon. In support of our finding, the cryo-EM data suggest that the N-terminal domain of eIF5 displaces eIF1 from the P-site of the 48S complex during AUG codon recognition. It is suggested that the eIF5^G31R^ mutation may prematurely displace eIF1 while scanning the mRNA from 5′ to 3′ direction and stabilizing the base pairing of Met-tRNAi anticodon with the UUG codon^4^. Furthermore, the ^35^S-Methionine labelling experiment suggested a less than 10% reduction in the protein biosynthesis in the eIF5^G31R^ mutant cells (data not shown). This subtle decrease in the protein translation may be an effect of oxidative stress in the eIF5^G31R^ mutant, as oxidative stress increases ribosomal transition time on mRNA and shows an inhibitory effect on translation elongation^55^. Consistently, we observed lower polysomes in the eIF5^G31R^ mutant (Supplementary Fig. S2).

Earlier in-vitro biochemical data suggest that the eIF5^G31R^ mutation disfavors the AUG base pairing while favors UUG base pairing in the ‘Closed/P_IN_’ conformation with the Met-tRNA_i_^56,57^, suggesting a possible mechanism of UUG codon recognition. However, it is not clear whether the eIF5^G31R^ mutant also disfavors AUG codon recognition in-vivo. Our data can resolve this issue; as upon removal of the upUUG codons from the *URA6-LacZ* or *YAP1-LacZ* reporter constructs, the levels of reporter expression initiated from the AUG codon in the eIF5^G31R^ mutant and WT cells were essentially identical (Figs. 2b, 4c), suggesting that the eIF5^G31R^ mutant can recognize AUG start codon in-vivo. Thus the dominant slow-growth phenotype and the recessive lethality could be because of the additional UUG codon recognition and the attendant proteome change in the eIF5^G31R^ mutant. The eIF2γ^N135D^ mutation confers a Sui^−^ phenotype, a slow-growth phenotype, and Met-tRNA_i_ binding defects that are consistent with its Gcd^−^ phenotype (de-repression of GCN4 expression). However, the slow-growth phenotype of the eIF2γ^N135D^ mutations is suppressed by the overexpression of Met-tRNA_i_ but not by the eIF1 overexpression, which only suppresses the Sui^−^ phenotype^17^. Taken together, it appears that the slow growth phenotype of the eIF2γ^N135D^ mutation is because of the TC formation defects. However, the dominant slow growth phenotype in the eIF5^G31R^ mutant is primarily because of the UUG codon recognition (Sui^−^ phenotype) and the attendant proteome change that causes oxidative stress and DNA damage, as the eIF5^G31R^ mutation does not have TC formation defect and show Gcn^−^ phenotype^19^. However, we do not imply that oxidative stress and DNA damage are the only defects caused by the upUUG codon recognition in the eIF5^G31R^ mutant cells. It could be possible that the mRNA transcript having multiple out of frame upUUG codons (examples, *URA6* or *YAP1*) may be disproportionately affected compared to the mRNA transcript having a single out of frame upUUG codon. Moreover, not all upUUG codons can be utilized efficiently to initiate translation by the Sui^−^ mutants as the sequence context surrounding the upUUG codon may also play an important role^58^. Consistently, the eIF5^G31R^ mutation showed ~ 2.6-fold decrease in UUG codon recognition efficacy when the UUG codon is in a poor sequence context (Supplementary Fig. S7). Probably genome-wide ribosomal profiling of the Sui^−^ mutants will give more clarity on global upUUG codon utilization in different sequence contexts and identification of affected proteins involved in various cellular pathways.

The significance of studying defects in the translation initiation process is that it uncovers the fundamentals of the information decoding process and sheds light on the diseases associated with this process. Diseases such as Alzheimer’s, amyotrophic lateral sclerosis, Vanishing White Matter (VWM) are linked with the defects associated with integrated stress response (ISR) that lower TC levels^59^. Mutations isolated in the human eIF2γ subunit are associated with the X-linked intellectual disability characterized by mental deficiency, epilepsy, hypogenitalism, microcephaly, and obesity (MEHMO syndrome)^60–63^. Interestingly, the yeast model of MEHMO syndrome mutations shows reduced TC levels and increased translation initiation at non-AUG codon. It is suggested that the difference in VWM disease and MEHMO syndrome may be due to the altered cellular proteome caused by the non-AUG codon initiation in the MEHMO syndrome patients^60^. Thus our study on translation initiation fidelity defective eIF5^G31R^ mutant that uncovered DNA damage and oxidative stress due to altered proteome could serve as an impetus to investigate further the proteome change and the attendant cellular defects caused by the eIF2γ mutants in the MEHMO syndrome patients.

## Supplementary Information

Supplementary Information.

## References

[1] Hinnebusch AG (2011). Molecular mechanism of scanning and start codon selection in eukaryotes. Microbiol. Mol. Biol. Rev..

[2] Hinnebusch AG (2014). The scanning mechanism of eukaryotic translation Initiation. Annu. Rev. Biochem..

[3] Llácer JL (2015). Conformational differences between open and closed states of the eukaryotic translation initiation complex. Mol. Cell.

[4] Llácer JL (2018). Translational initiation factor eIF5 replaces eIF1 on the 40S ribosomal subunit to promote start-codon recognition. Elife.

[5] Fekete CA (2005). The eIF1A C-terminal domain promotes initiation complex assembly, scanning and AUG selection in vivo. EMBO J..

[6] Alone PV, Dever TE (2006). Direct binding of translation initiation factor eIF2gamma-G domain to its GTPase-activating and GDP-GTP exchange factors eIF5 and eIF2B epsilon. J. Biol. Chem..

[7] Maag D, Fekete CA, Gryczynski Z, Lorsch JR (2005). A conformational change in the eukaryotic translation preinitiation complex and release of eIF1 signal recognition of the start codon. Mol. Cell.

[8] Chang K-J, Wang C-C (2004). Translation initiation from a naturally occurring non-AUG codon in *Saccharomyces cerevisiae*. J. Biol. Chem..

[9] Chen S-JJ, Lin G, Chang K-JJ, Yeh L-SS, Wang C-CC (2008). Translational efficiency of a non-AUG initiation codon is significantly affected by its sequence context in yeast. J. Biol. Chem..

[10] Iwasaki S, Floor SN, Ingolia NT (2016). Rocaglates convert DEAD-box protein eIF4A into a sequence-selective translational repressor. Nature.

[11] Guenther UP (2018). The helicase Ded1p controls use of near-cognate translation initiation codons in 5′ UTRs. Nature.

[12] Costello J (2015). Global mRNA selection mechanisms for translation initiation. Genome Biol..

[13] Mitchell SF (2010). The 5’-7-methylguanosine cap on eukaryotic mRNAs serves both to stimulate canonical translation initiation and to block an alternative pathway. Mol. Cell.

[14] Gupta N, Lorsch JR, Hinnebusch AG (2018). Yeast Ded1 promotes 48S translation preinitiation complex assembly in an mRNA-specific and eIF4F-dependent manner. Elife.

[15] Castilho-Valavicius B, Yoon H, Donahue TF (1990). Genetic characterization of the *Saccharomyces cerevisiae* translational initiation suppressors *sui1, sui2* and *SUI3* and their effects on *HIS4* expression. Genetics.

[16] Huang HK, Yoon H, Hannig EM, Donahue TF (1997). GTP hydrolysis controls stringent selection of the AUG start codon during translation initiation in *Saccharomyces cerevisiae*. Genes Dev..

[17] Alone PV, Cao C, Dever TE (2008). Translation initiation factor 2gamma mutant alters start codon selection independent of Met-tRNA binding. Mol. Cell. Biol..

[18] Antony AC, Ram AK, Dutta K, Alone PV (2019). Ribosomal mutation in helix 32 of 18S rRNA alters fidelity of eukaryotic translation start site selection. FEBS Lett..

[19] Antony AC, Alone PV (2017). Defect in the GTPase activating protein (GAP) function of eIF5 causes repression of GCN4 translation. Biochem. Biophys. Res. Commun..

[20] Antony AC, Alone PV (2018). Fidelity of HIS4 start codon selection influences 3-amino-1,2,4-triazole sensitivity in GTPase activating protein function defective eIF5. J. Genet..

[21] Gietz RD, Sugino A, Akio S (1988). New yeast-*Escherichia coli* shuttle vectors constructed with in vitro mutagenized yeast genes lacking six-base pair restriction sites. Gene.

[22] Gueldener U, Heinisch J, Koehler GJ, Voss D, Hegemann JH (2002). A second set of loxP marker cassettes for Cre-mediated multiple gene knockouts in budding yeast. Nucleic Acids Res..

[23] Christianson TW, Sikorski RS, Dante M, Shero JH, Hieter P (1992). Multifunctional yeast high-copy-number shuttle vectors. Gene.

[24] Perez-Riverol Y (2019). The PRIDE database and related tools and resources in 2019: Improving support for quantification data. Nucleic Acids Res..

[25] Tkach JM (2012). Dissecting DNA damage response pathways by analysing protein localization and abundance changes during DNA replication stress. Nat. Cell Biol..

[26] Uttenweiler A, Schwarz H, Neumann H, Mayer A (2007). The vacuolar transporter chaperone (VTC) complex is required for microautophagy. Mol. Biol. Cell.

[27] Jager S, Strayle J, Heinemeyer W, Wolf DH (2001). Cic1, an adaptor protein specifically linking the 26S proteasome to its substrate, the SCF component Cdc4. EMBO J..

[28] Kumánovics A (2008). Identification of *FRA1* and *FRA2* as genes involved in regulating the yeast iron regulon in response to decreased mitochondrial iron-sulfur cluster synthesis. J. Biol. Chem..

[29] Goder V, Melero A (2011). Protein O-mannosyltransferases participate in ER protein quality control. J. Cell Sci..

[30] Altmann M, Schmitz N, Berset C, Trachsel H (1997). A novel inhibitor of cap-dependent translation initiation in yeast: p20 competes with eIF4G for binding to eIF4E. EMBO J..

[31] Sertil O, Kapoor R, Cohen BD, Abramova N, Lowry CV (2003). Synergistic repression of anaerobic genes by Mot3 and Rox1 in *Saccharomyces cerevisiae*. Nucleic Acids Res..

[32] Vergauwen B, Pauwels F, Van Beeumen JJ (2003). Glutathione and catalase provide overlapping defenses for protection against respiration-generated hydrogen peroxide in *Haemophilus influenzae*. J. Bacteriol..

[33] Malys N, McCarthy JEG (2006). Dcs2, a novel stress-induced modulator of m7G pppX pyrophosphatase activity that locates to P bodies. J. Mol. Biol..

[34] Skoneczna A, Miciałkiewicz A, Skoneczny M (2007). *Saccharomyces cerevisiae* Hsp31p, a stress response protein conferring protection against reactive oxygen species. Free Radic. Biol. Med..

[35] Fakas S (2017). Lipid biosynthesis in yeasts: A comparison of the lipid biosynthetic pathway between the model nonoleaginous yeast *Saccharomyces cerevisiae* and the model oleaginous yeast *Yarrowia lipolytica*. Eng. Life Sci..

[36] Pallotta ML, Valenti D, Iacovino M, Passarella S (2004). Two separate pathways for d-lactate oxidation by *Saccharomyces cerevisiae* mitochondria which differ in energy production and carrier involvement. Biochim. Biophys. Acta BBA Bioenergy.

[37] Lagoskyl PA, Taylor GR, Haynes RH (1987). Molecular characterization of the S*accharomyces cerevisiae* dihydrofolate reductase gene (DFRI). Nucleic Acids Res..

[38] Huang Z (2018). OLE1 reduces cadmium-induced oxidative damage in *Saccharomyces cerevisiae*. FEMS Microbiol. Lett..

[39] Grishin AV, Rothenberg M, Downs MA, Blumer KJ (1998). Mot3, a Zn finger transcription factor that modulates gene expression and attenuates mating pheromone signaling in *Saccharomyces cerevisiae*. Genetics.

[40] Zhang S, Sanyal I, Bulboaca GH, Rich A, Flint DH (1994). The gene for biotin synthase from *Saccharomyces cerevisiae*: Cloning, sequencing, and complementation of *Escherichia coli* strains lacking biotin synthase. Arch. Biochem. Biophys..

[41] García-Ramírez JJ, Santos MA, Revuelta JL (1995). The *Saccharomyces cerevisiae RIB4* gene codes for 6,7-dimethyl-8-ribityllumazine synthase involved in riboflavin biosynthesis. J. Biol. Chem..

[42] Fatica A, Oeffinger M, Tollervey D, Bozzoni I (2003). Cic1p/Nsa3p is required for synthesis and nuclear export of 60S ribosomal subunits. RNA.

[43] Ogg SC, Poritz MA, Walter P (1992). Signal recognition particle receptor is important for cell growth and protein secretion in *Saccharomyces cerevisiae*. Mol. Biol. Cell.

[44] McNew JA (1998). Gos1p, a *Saccharomyces cerevisiae* SNARE protein involved in Golgi transport. FEBS Lett..

[45] Jiang ZR, Abaigar LT, Huang SH, Cai B, Jong AY (1991). Molecular characterization of *Saccharomyces cerevisiae URA6* gene. DNA sequence, mutagenesis analysis, and cell cycle regulation relevant to its suppression mechanism to cdc8 mutation. J. Biol. Chem..

[46] Patricia L, Francois L (1986). Genetic characterization and isolation of the *Saccharomyces cerevisiae* gene coding for uridine monophosphokinase. Mol. Gen. Genet..

[47] Jamieson DJ (1998). Oxidative stress responses of the yeast *Saccharomyces cerevisiae*. Yeast Chichester Engl..

[48] Kurutas EB (2016). The importance of antioxidants which play the role in cellular response against oxidative/nitrosative stress: Current state. Nutr. J..

[49] He L (2017). Antioxidants maintain cellular redox homeostasis by elimination of reactive oxygen species. Cell. Physiol. Biochem. Int. J. Exp. Cell. Physiol. Biochem. Pharmacol..

[50] Machado MD, Soares EV (2012). Assessment of cellular reduced glutathione content in *Pseudokirchneriella subcapitata* using monochlorobimane. J. Appl. Phycol..

[51] Bankapalli K (2015). Robust glyoxalase activity of Hsp31, a ThiJ/DJ-1/PfpI family member protein, is critical for oxidative stress resistance in *Saccharomyces cerevisiae*. J. Biol. Chem..

[52] Perrone GG, Tan SX, Dawes IW (2008). Reactive oxygen species and yeast apoptosis. Biochim. Biophys. Acta Mol. Cell Res..

[53] Lee J, Spector D, Godon C, Labarre J, Toledano MB (1999). A new antioxidant with alkyl hydroperoxide defense properties in yeast. J. Biol. Chem..

[54] Salmon TB, Evert BA, Song B, Doetsch PW (2004). Biological consequences of oxidative stress-induced DNA damage in *Saccharomyces cerevisiae*. Nucleic Acids Res..

[55] Shenton D (2006). Global translational responses to oxidative stress impact upon multiple levels of protein synthesis. J. Biol. Chem..

[56] Saini AK (2014). Eukaryotic translation initiation factor eIF5 promotes the accuracy of start codon recognition by regulating Pi release and conformational transitions of the preinitiation complex. Nucleic Acids Res..

[57] Martin-Marcos P (2014). Enhanced eIF1 binding to the 40S ribosome impedes conformational rearrangements of the preinitiation complex and elevates initiation accuracy. RNA.

[58] Martin-Marcos P, Cheung Y-N, Hinnebusch AG (2011). Functional elements in initiation factors 1, 1A, and 2β discriminate against poor AUG context and non-AUG start codons. Mol. Cell. Biol..

[59] Costa-Mattioli M, Walter P (2020). The integrated stress response: From mechanism to disease. Science.

[60] Young-Baird SK (2020). Suppression of MEHMO syndrome mutation in eIF2 by small molecule ISRIB. Mol. Cell.

[61] Young-Baird SK, Shin B-S, Dever TE (2019). MEHMO syndrome mutation *EIF2S3-I259M* impairs initiator Met-tRNAiMet binding to eukaryotic translation initiation factor eIF2. Nucleic Acids Res..

[62] Skopkova M (2017). EIF2S3 mutations associated with severe X-linked intellectual disability syndrome MEHMO. Hum. Mutat..

[63] Borck G (2012). eIF2γ mutation that disrupts eIF2 complex integrity links intellectual disability to impaired translation initiation. Mol. Cell.

[64] Krzywinski M (2009). Circos: An information aesthetic for comparative genomics. Genome Res..

