# Supplementary material for: Altered proteome in translation initiation fidelity defective eIF5G31R mutant causes oxidative stress and DNA damage
Source: Sci Rep. 2022 Mar 23;12:5033. doi: 10.1038/s41598-022-08857-y (PMC8943034; doi:10.1038/s41598-022-08857-y)
Supplement: Supplementary file 1 — Supplementary Information. [file 41598_2022_8857_MOESM1_ESM.pdf]

Altered proteome in translation initiation fidelity defective eIF5^{G31R} mutant causes oxidative stress and DNA damage

Anup Kumar Ram[1,2], Monalisha Mallik[1,2], R. Rajendra Reddy[3], Amol Ratnakar Suryawanshi[3] and Pankaj V. Alone[1,2]

Supplementary data

Supplementary Table 1: Oligonucleotides used in this study

Sr. No	Oligo-nucleotide	Sequence (5′-3′)
1	oPA1005	CACCAAGCTTTAATGCGTCATCATCCCAACTTAACACCCCTCATATACCC
2	oPA1006	CGCAGTCGACGAAAGCTGGCTGTGATGTAGTGGCAGCTGTCATTTGTCTT
3	oPA1007	CACCGTCGACGTCGTTTTACAACGTCGTGACTGGGAAAAC
4	oPA1008	CGCAGGATCCTTATTTTTGACACCAGACCAACTGGTAATGGTAGCGACCG
5	oPA1009	CACCGGATCCACTAAGACGTATTTTATATAGTTTACGCTATAAGTTACGGCGA
6	oPA1010	CGCAGGTACCGTTAAGGAGTCACTACCGACTGT
7	oPA1011	CGCAGTCGACGAAAGCTGGCTGTGATGTAGTGGCAGCTGTCATTTGTCTTATTCGAGAAATTCGACTGGTTCGTTCTTTTG
8	oPA1012	AGTGCTAAAGTTCCTACGAGTACCGACGATTTGCCTTTGTGTTTA
9	oPA1013	GTACTCGTAGGAACTTTAGCACTCGTGACATCAGTTATTTCGGTA
10	oPA1034	CGCAAAGCTTCCAGAGATGATGAGGAACCGATGACGAGGCACATGGGGAT
11	oPA1036	CGCAGGATCCGGCAGCACTATTTATTCATAATTATTCGTAATTATATCTC

12	oPA1037	CGCAGGTACCACTTTGGTAAGATATTATTATAACAGAAAGAATTTAAAGT
13	oPA1040	CGCAGGATCCATTAGCGTAATCTGGAACATCGTATGGGTAAGCGTAATCTGGAACATCGTATGGGTAAGCGTAATCTGGAACATCGTATGGGTAATTGGCACTTGCAATGGAC
14	oPA1041	CGCAGGATCCCTAAGCGTAATCTGGAACATCGTATGGGTAAGCGTAATCTGGAACATCGTATGGGTAAGCGTAATCTGGAACATCGTATGGGTATAAGCTATCACGGATAGCGT
15	oPA1042	CGCAGTCGACTCGCCTGACCAAGTTTCCGTGATCTTCGTTCTAGGAGGAC
16	oPA1089	CGCAGTCGACGTTAAGGAGTCACTACCGAC
17	oPA1101	CTATTGGAGCGTGGCAAGAC
18	oPA1102	AACGAACACGGACGACTTTC
19	oPA1108	CGCAGCGGCCGCCCAGAGATGATGAGGAACCGATGAC
20	oPA1109	CGCAGGATCCGTAAGATATTATTATAACAGAAAGA
21	oPA1113	AAAAAGAGAGTTACGCAATA
22	oPA1114	AACATCGTATGGGTAATTGG
23	oPA1173	CGCAGGATCCACCTAATTAGACATGGCATG
24	oPA1174	CGCAGCGGCCGCACACTACTAGCTCCTGAAGA
25	oPA1175	CGCAAAGCTTAGGTCAAGAAAAGTATATGC
26	oPA1176	CGCAGGATCCATTTTGCTGTGCCCCCGGTA
27	oPA1467	CACCGTCGACTGATGTAGTGGCAGCTGTCGTTTGTCTATATTCGAGAAATTCGACTGG
28	oPA1468	GTTCCTACGAGTACCGACATTTTGCCTTTGTGTTTACCCT
29	oPA1469	AAGGCAAAATGTCGGTACTCGTAGGAACTTTAGCA

30	oPA1470	CACCGTCGACTGATGTAGTGGCAGCTGTCGTTGTCTTATTCAAGAAATTCAA
31	oPA1471	CACCGTCGACTGATGTAGTGGCAGCTGTCGTTGTCTTATTCAAGAAATTCAA
32	oPA1534	ATGAACGTGTTCGGTAAAAAAGAAGAAAAGCAAGAAAAAGCAGCTGAAGCTTCGTACGC
33	oPA1535	TTAATTGGCACTTGCAATGGACCAAGTCTTGGCATAACCTCGCATAGGCCACTAGTGGAT
34	oPA1559	CGCAGGATCCATTACGGATTCACTGGCCGTCGTTTTACAACGT
35	oPA1560	CGCAGTCGACTTATTTTTGACACCAGACCA
36	oPA1569	TATTTTCGACGTTGAAGA
37	oPA1607	CGCAACTAGTTTTGTATTTATCGGAAACG
38	oPA1608	CGCAAAGCTTGAGTTCCATCAATGCTATGA
39	oPA1609	CGCAGGATCCCCTCTTGGCGGTAGACACACTCATGGTTTAAGA
40	oPA1610	CGCAGTCGACAGCGGGAACTTTATGGAAAA
41	oPA1636	GATCGGTGTTTAGCTTTTTTTC
42	oPA1637	AAAAAGCTAAACACCGATCCTATATATAACGTAAA
43	oPA1638	ACTCGTGCATGAACACGAGCCA
44	oPA1639	CTCGTGTTCATGCACGAGTCGACCAGGGGCTCAGGAAAA
45	oPA1640	CGCAGGATCCCTTGGCGGTAGACACACTCATGGTTTAAGAAACGACTTTTCCTTCTTTAAACGTTTTGGGTGGCGAAAAACTTCCCTTAAACGAACTAAAAATGGCTCGTG
46	oPA1641	CAGAGCTTCCGAAACAATAT
47	oPA1642	TTTCGGATGTGTTGTTATTC
48	oPA1024	TTAGAAACACTTGTGGTGAACGATAG
49	oPA1023	ATGGATTCTGAGGTTGCTGCTTTGG

50	oPA650	CAATTCGTTGTAGAAGGTATGATGCC
51	oPA584	TTGGCTAATCATGACCCC
52	oPA1754	GTGCTTTCACCAAATCCATT
53	oPA1755	AAGACACTTTCGACTGAGGA
54	oPA1340	CGCAGCATGCCTCATCGGAAGAGGTGGCAT
55	oPA1341	CGCAGGATCCATTATTTCTAACTTGGAAAC
56	oPA1342	CGCAGGTACCGTCAGACTCGTTGGAATTTG
57	oPA1343	CGCAGTCGACCGGTAGAATCGGCATTTTCGTTATTCAGAAAAAAAT TTTGT
58	oPA1344	CGCAGTCGACCGGTAGAATCGGCATAAACGTTATTCAGAAAAAAAT TTTGT
59	oPA1345	CGCAGTCGACCGGTAGAATCGGCAATTTCGTTATTCAGAAAAAAAT TTTGT
60	oPA1346	CGCAGTCGACCGGTAGAATCGGCAAAAACGTTATTCAGAAAAAAAT TTTGT

Supplementary Table 2. Plasmids used in this study

Sr. No.	Plasmid Number	Plasmid Name	Type	Reference
1	A1361	pYcplac33-His4-AAA-AUG-lacZ	s.c	This Study
2	A1362	pYcplac33-His4-UUU-AUG-lacZ	s.c	This Study
3	A1363	pYcplac33-His4-AAA-UUG-lacZ	s.c	This Study
4	A1364	pYcplac33-His4-UUU-UUG-lacZ	s.c	This Study

Supplementary Table 3: Classification of altered proteins based on their involvement in various biological processes

Sr. No	Biological process	Genes	Fold change ($eIF5^{G31R}$/WT)	P-value	Presence of UUG codon
1	Autophagy	VTC2	1.9087	0.0453	Yes
2		VTC3	1.7513	0.0068	Yes
3		VTC4	1.8045	0.0040	No
4	Lipid Biosynthesis	CHO1	0.6500	0.0246	Yes
5		OLE1	0.5477	0.0124	Yes
6	Cellular Stress	HSP12	1.8912	0.0252	No
7		HSP31	1.8778	0.0026	No
8		MOT3	3.3204	0.0459	Yes
9		CTT1	2.4573	0.0475	Yes
10		DCS2	2.2230	0.0340	Yes
11	Protein Catabolism	CIC1	0.6613	0.02	No
12		PMT2	1.5368	0.0344	No
13		FRA1	1.6199	0.0396	No
14	DNA Damage	HSP31	1.8778	0.0026	No
15		RGI1	1.6674	0.0044	No
16		HSP12	1.8912	0.0252	No
17		DUR1,2	1.7275	0.0011	Yes
18		VTC4	1.8045	0.0040	No
19		TOH1	1.7233	0.0489	No
20		CTF4	1.7217	0.0309	No
21	Translational Repression	CAF20	1.9685	0.0059	No
22		DCS2	2.2230	0.0340	Yes
23	Nucleotide Biosynthesis	URA6	0.5546	0.0069	Yes
24	Protein Transport	GOS1	2.3314	0.0344	No
25		SRP101	0.6570	0.0304	Yes
26	Ribosome Biogenesis	CIC1	0.6613	0.0200	No
27	Oxidation-Reduction Process	DLD1	0.6661	0.005	Yes
28		OLE1	0.5477	0.0124	Yes
29		DFR1	1.5977	0.0424	No
30		CTT1	2.4573	0.0475	Yes
31	Transcription	FRA1	1.6199	0.0396	No
32		MOT3	3.3204	0.0459	Yes
33	Vitamin/Co-factor Biosynthesis	RIB4	1.5247	0.0261	No
34		BIO2	0.6493	0.0081	No
35	Unknown Function	YNL208W	1.8591	0.0191	No
	Carbohydrate Metabolism	ENO2	0.3448	0.0456	Yes
		FBP1	0.6040	0.0401	Yes
			1.6507	0.0026	
36		PDC1	2.6654	0.0299	No
37	Hydrogen Peroxide/ Catabolic Process	AHP1	0.6047	0.0370	No

Methods

Construction of HIS4-LacZ reporter in good and poor sequence context: The pYcplac33-His4-lacZ reporter construct was generated as follows- first, the 541 bp of HIS4 promoter and initiator region with change in start codon to ACG followed by AAA_AUG, UUU_AUG, AAA_UUG, and UUU_UUG codons were introduced by PCR using reverse primer oPA1343, oPA1344, oPA1345 and oPA1346 respectively with forward primer oPA1340. The amplified PCR product was cloned into cloning vector pYcplac33 (A309) at SphI and SalI site. The LacZ region and His4 3′ UTR region were PCR amplified using oligonucleotides combination oPA1009/oPA1010 and oPA1341/oPA1342 respectively and cloned at SalI-BamHI and BamHI-KpnI sites.

Supplementary figure legends

Figure S1. Schematic of iTRAQ experiment. Three biological replicates of WT or eIF5^{G31R} mutant were grown till OD_{600} ~0.8 at 30°C. The total cell extract was subjected to trypsin digestions and labelled with iTRAQ 4-plex kit, subjected to cation exchange and reverse phase chromatography. The samples from set 1 and set 2 were subjected to MALDI/TOF/MS analysis (a) and the summary of iTRAQ data analysis is shown in (b).

Figure S2. Analysis of polysome profile. Yeast strain YP823 carrying an empty vector (EV) or eIF5^{G31R} mutation, were grown till OD_{600} ~0.8 at 30°C. The cell culture (200 ml) was treated 50 µg/ml cycloheximide for 5 min before harvesting. Whole cell extract (OD_{254} ~ 20) was resolved on 5–45% sucrose density gradient using BIOCOMP gradient station. OD_{254} versus time (*t*) was plotted. The 40S, 60S, and 80S peaks are indicated with black, gray, and white arrowheads, respectively. Polysome to monosome ration (P/M) for each sample is indicated.

Figure S3. Full-membrane images of *URA6* immunoblots and full-agarose gel image of *URA6* real-time PCR product presented in Figure 2a.

Figure S4. Full-membrane images of *CTT1* immunoblots and full-agarose gel image of *CTT1* real-time PCR product presented in Figure 3a.

Figure S5. Full-agarose gel image of *YAP1* real-time PCR product presented in Figure 4c.

Figure S6. Real time PCR for *AHP1* and full-agarose gel image of real-time PCR product. YP823 strain carrying empty vector pYcplac22 (A823) or pYcplac22-eIF5^{G31R} (A838) constructs were grown overnight on synthetic dextrose (SD) plus leucine & uracil medium till OD_{600} ~0.8 at 30°C. The whole-cell extract (WCE) was treated with TRIzol, followed by ethanol precipitation. The total RNA isolated was subjected to reverse transcription and quantitative PCR using *AHP1* ORF specific

oligonucleotides oPA1754 and oPA1755. The error represents an average deviation. Statistical differences were determined by the two-tailed Student's t-test.

Figure S7. Analysis of HIS4-LacZ expression. Yeast strain YP823 containing empty vector pYcplac22 (A823) or pYcplac22-eIF5^{G31R} (A838) constructs were transformed with derivatives of His4-LacZ reporter constructs containing AUG codon in a good (A1361) and poor (A1362) sequence context, or UUG codon in a good (A1363) and poor (A1364) sequence context, were grown till OD_{600} ~ 0.8 in SD plus leucine medium at 30°C. WCE were prepared, and β-galactosidase activity (nmol of O-nitrophenyl-β-D-galactopyranoside cleaved per min per mg) was measured and the ratio of poor/good sequence context is plotted. The error represents an average deviation. Statistical differences were determined by the two-tailed Student's t-test.

a

Schematic of the iTRAQ experiment

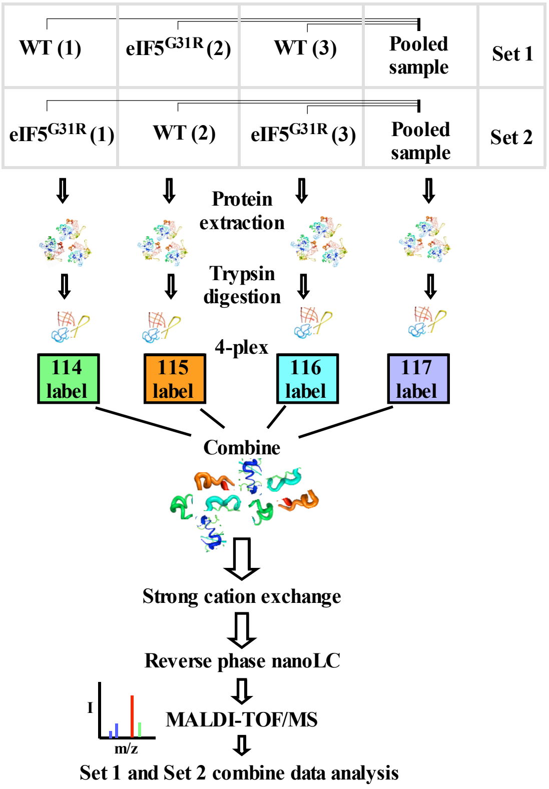

b

Summary of iTRAQ data analysis

		Local FDR 5%		Global FDR 5%	
		Set 1	Set 2	Set 1	Set 2
1	Number of spectra	19775	17341	22837	20467
2	Number of distinct peptides	11523	10277	14461	13024
3	Number of proteins identified	1491	1348	1603	1464
4	Significant protein (unused score ≥ 1.3)			1436	1298
5	Proteins common in both the sets			1098	
6	Significant (P-value ≤ 0.05)			154	
7	Significantly altered proteins (cut-off >1.5 and < 0.66; P-value ≤ 0.05)			19 up-regulated<br>7 down-regulated	

Figure: S1

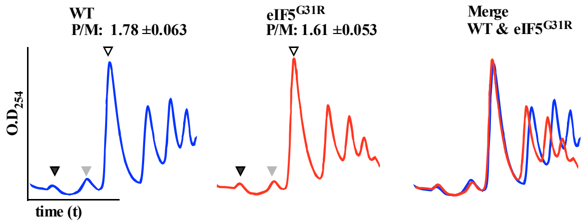

Figure: S2

Supplementary figures

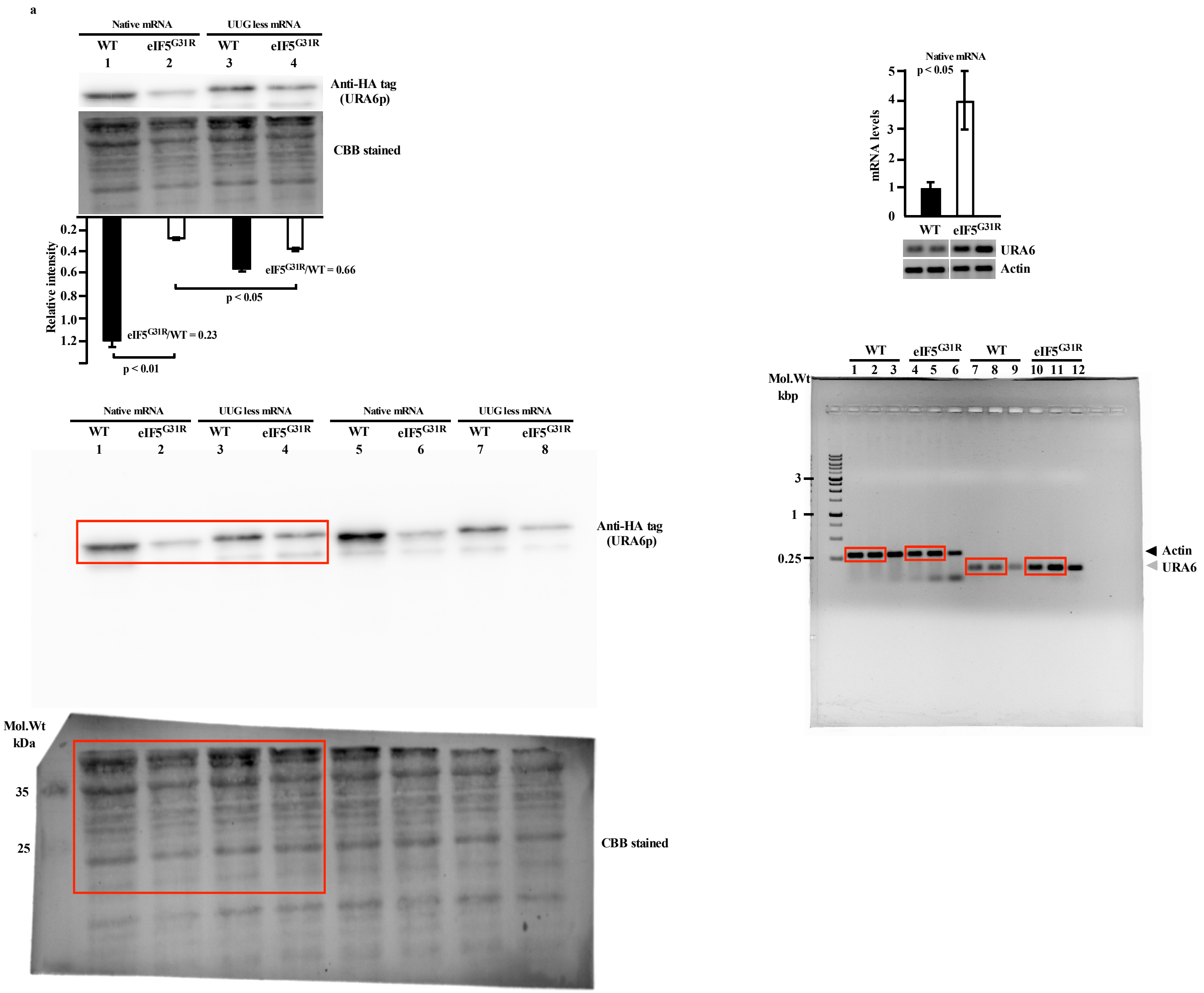

Figure: S3

Supplementary figures

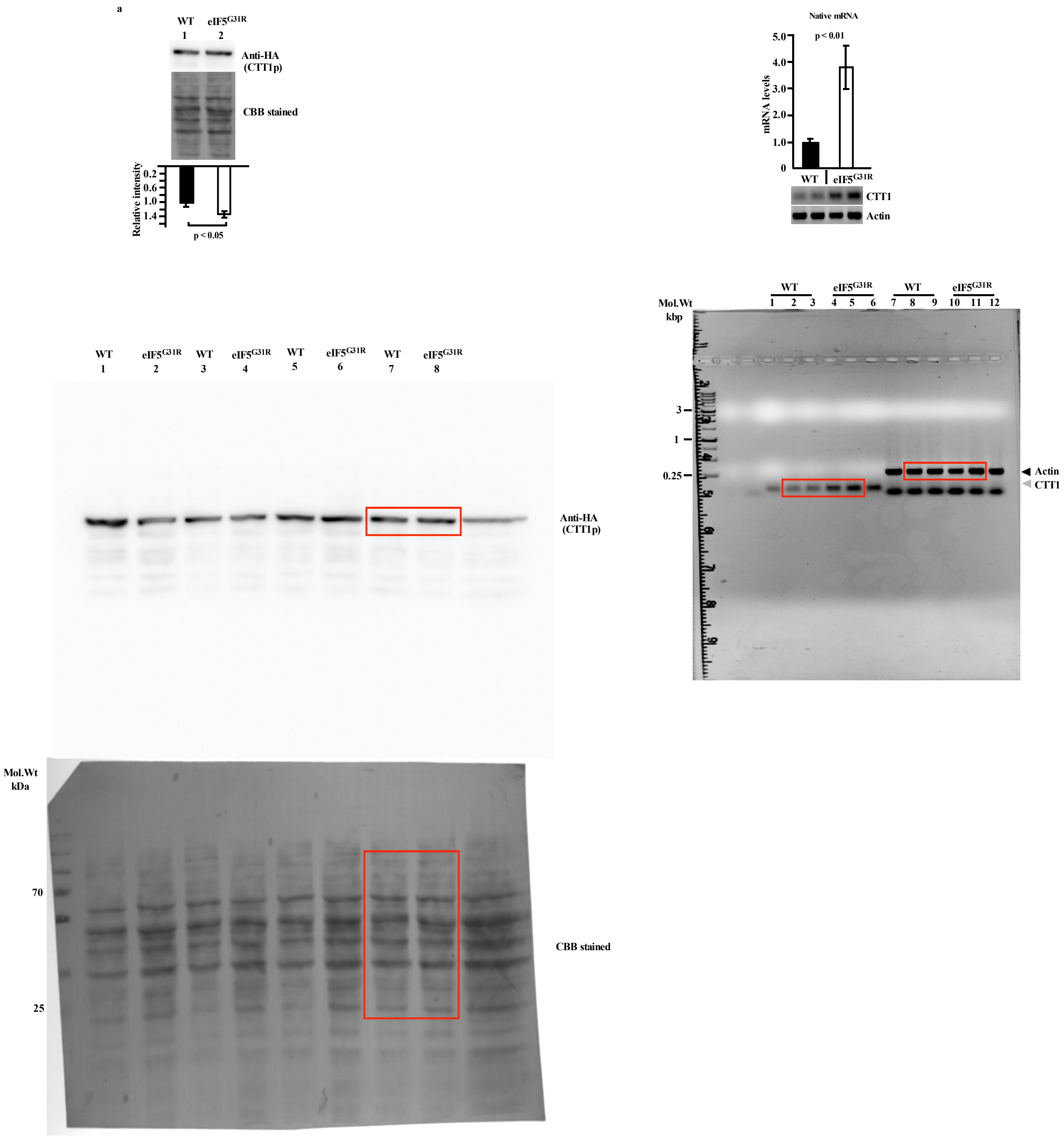

Figure: S4

Supplementary figures

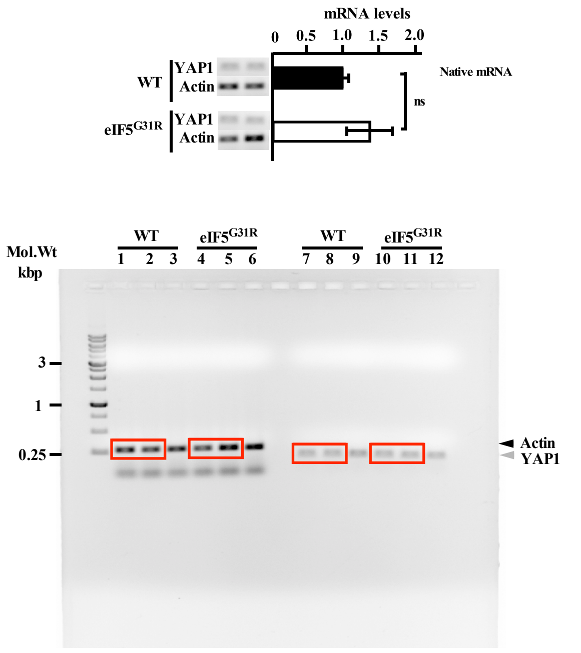

Figure: S5

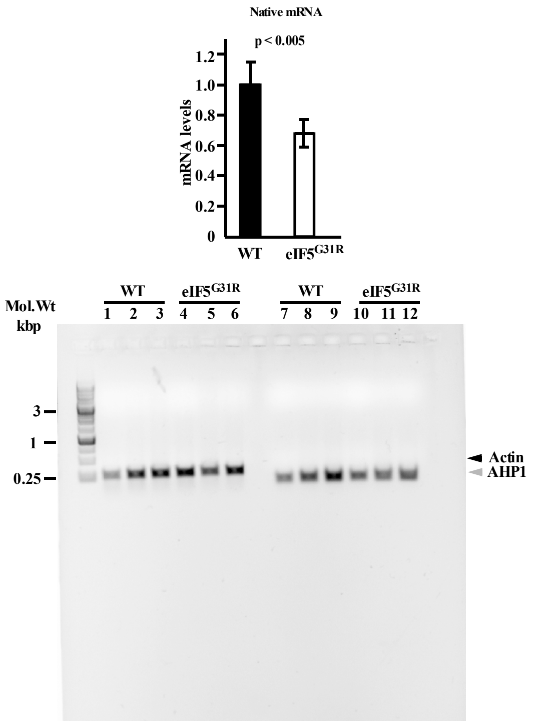

Figure: S6

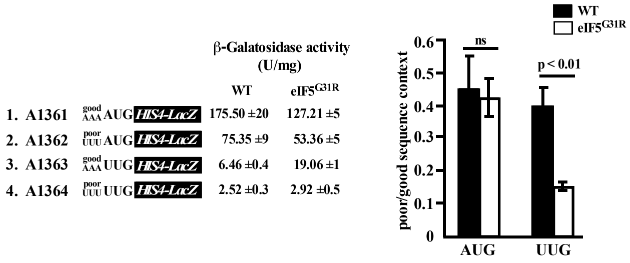

Figure: S7

Supplementary figures
